# Phage typing or CRISPR typing for epidemiological surveillance of *Salmonella* Typhimurium?

**DOI:** 10.1186/s13104-017-2878-0

**Published:** 2017-11-07

**Authors:** Manal Mohammed

**Affiliations:** Department of Biomedical Sciences, Faculty of Science and Technology, University of Westminster, London, UK

**Keywords:** *Salmonella* Typhimurium, CRISPR typing, Phage typing, Surveillance, Outbreaks

## Abstract

**Objective:**

*Salmonella* Typhimurium is the most dominant *Salmonella* serovar around the world. It is associated with foodborne gastroenteritis outbreaks but has recently been associated with invasive illness and deaths. Characterization of *S.* Typhimurium is therefore very crucial for epidemiological surveillance. Phage typing has been used for decades for subtyping of *S.* Typhimurium to determine the epidemiological relation among isolates. Recent studies however have suggested that high throughput clustered regular interspaced short palindromic repeats (CRISPR) typing has the potential to replace phage typing. This study aimed to determine the efficacy of high-throughput CRISPR typing over conventional phage typing in epidemiological surveillance and outbreak investigation of *S.* Typhimurium.

**Results:**

In silico analysis of whole genome sequences (WGS) of well-documented phage types of *S.* Typhimurium reveals the presence of different CRISPR type among strains belong to the same phage type. Furthermore, different phage types of *S.* Typhimurium share identical CRISPR type. Interestingly, identical spacers were detected among outbreak and non-outbreak associated DT8 strains of *S.* Typhimurium. Therefore, CRISPR typing is not useful for the epidemiological surveillance and outbreak investigation of *S.* Typhimurium and phage typing, until it is replaced by WGS, is still the gold standard method for epidemiological surveillance of *S.* Typhimurium.

**Electronic supplementary material:**

The online version of this article (10.1186/s13104-017-2878-0) contains supplementary material, which is available to authorized users.

## Introduction

Salmonellosis is one of the most common causes of foodborne disease worldwide. Nontyphoidal salmonellosis (NTS) is a zoonotic disease transmitted from animals to humans through consumption of contaminated food. Worldwide, *Salmonella enterica* serovar Typhimurium (*S.* Typhimurium) accounts for most human infection of NTS and has been associated with foodborne outbreaks in developing and developed countries resulting in high morbidity and mortality [1]. Furthermore, the recent emergence of the multidrug-resistant (MDR) *S.* Typhimurium variant of a distinct Sequence Type ST313 in sub-Saharan Africa represents a major public health concern as it is associated with invasive illness and deaths [2]. An efficient laboratory system for epidemiological surveillance and outbreak investigation of *Salmonella* Typhimurium is therefore very crucial.

Phage typing system is a phenotypical method that has been used for decades for subtyping of *S.* Typhimurium to determine the epidemiological relation among isolates [3]. Phage typing is a rapid and low cost approach for the epidemiological surveillance and outbreak investigation of *S.* Typhimurium. The system distinguishes more than 300 definitive phage types (DT) of *S.* Typhimurium based on their patterns of lysis to a unique collection of *Salmonella* phages but it has shown some limitations including the maintenance of typing phages by the reference laboratory and the updating of the system furthermore it depends entirely on the experience of the individual laboratory for interpretation of the results [4].

Recent studies have suggested that high throughput clustered regular interspaced short palindromic repeats (CRISPRs) typing and the microbead-based CRISPOL assay have the potential to replace traditional bacterial typing and subtyping systems including phage typing [5, 6]. CRISPRs consist of direct repeats (DRs) separated by variable spacer sequences that are derived from foreign phages or plasmids [7] while CRISPOL is a bead-based liquid hybridization assay for CRISPR polymorphism [5].

A recent study reported identical CRISPRs between two different phage types of *S.* Typhimurium; DT8 and DT30 [8] which reveals the limitations of CRISPR typing for epidemiological surveillance of *S.* Typhimurium.

This study aimed to analyze the CRISPR/CRISPOL type of well-documented phage types of *S.* Typhimurium in order to determine the efficacy of high-throughput CRISPR and CRISPOL typing over conventional phage typing in epidemiological surveillance of *S.* Typhimurium.

## Main text

### Methods

#### Whole genome sequence of different phage types of *S.* Typhimurium

The whole genome sequence of well-documented phage types of *S.* Typhimurium (Tables 1, 2) were obtained from Enterobase (https://enterobase.warwick.ac.uk/). Furthermore, a set of different phage types of *S.* Typhimurium that are used as control in Anderson phage typing scheme (Tables 1, 2) were selected for whole genome sequencing (WGS). Genomic DNA was extracted using QIAamp DNA Mini Kit (Qiagen) according to manufacturer’s instructions and submitted for WGS using an Illumina MiSeq on 250 bp paired-end (PE) libraries. The quality of PE data was evaluated using FastQC toolkit (http://www.bioinformatics.babraham.ac.uk/projects/fastqc/). Adapter sequences were removed using ea-utils package (https://expressionanalysis.github.io/ea-utils/). PE reads for each isolate were de novo assembled using velvet [9]. The best assembly with the highest N50 value was obtained. Raw sequence data of control phage types of *S.* Typhimurium have been submitted to the European Nucleotide Archive (ENA) under study Accession No.: PRJEB18673 (http://www.ebi.ac.uk/ena/data/view/PRJEB18673) and also available via Enterobase (https://enterobase.warwick.ac.uk/).

**Table 1:** *Salmonella* Typhimurium strains belonging to the same phage type show different CRISPR/CRISPOL type

Phage type	Isolate ID (source)	Lab	Accession Number	CRISPR type	CRISPOL type	References
DT1	DT1 (Clinical isolate)	Wellcome Trust Sanger Institute	^a^Traces-0ajzxba (ERS007598)	*8579*	*430*	[2]
	TM 68-619 (Clinical isolate)	Institut Pasteur	Traces-0MviFiU	*2536*	*54*	Enterobase
	TM 65-111 (Clinical isolate)	Institut Pasteur	Traces-0BvXZSr	*7387*	*90*	Enterobase
DT10	MS34 (Control DT10)	NSSLRL	^b^Traces-0eeFHtx (PRJEB18673)	*9509*	*1629*	This study
	S81-784 (Clinical isolate)	Institut Pasteur	Traces-0bXCHix	*9913*	*1688*	Enterobase
DT15a	MS41 (Control DT15a)	NSSLRL	^b^Traces-0FVsVub (PRJEB18673)	*9517*	*1634*	This study
	S81-798 (Clinical isolate)	Institut Pasteur	Traces-0QWCSHz	*9916*	*1756*	Enterobase
DT41	M11-2004 (Control DT41)	NSSLRL	^b^Traces-0hioJez (PRJEB18673)	*9513*	*1630*	This study
	CQ 41 (Clinical isolate)	Institut Pasteur	Traces-0BkvapO	*7434*	*223*	Enterobase
	S02-0321 (Clinical isolate)	Institut Pasteur	Traces-0JWTeTs	*9929*	*1766*	Enterobase

^a^Accession Numbers in Enterobase of clinical isolates of *S.* Typhimurium used in this study. The Accession Number in ENA for each isolate is also provided

^b^Accession Numbers in Enterobase of control phage types of *S.* Typhimurium sequenced in this study. The Accession Number in ENA is also provided

**Table 2:** *Salmonella* Typhimurium strains belonging to different phage types show identical CRISPR/CRISPOL type

Phage type	Isolate ID (source)	Lab	Accession Number	CRISPR type	CRISPOL type	Reference
CRISPR/CRISPOL type among phage types DT8 and DT30 of *S.* Typhimurium						
DT8	M18-2003 (Control DT8)	NSSLRL	^a^Traces-0jdDfGp (PRJEB18673)	*1069*	*6*	This study
DT8	DT8 (Clinical isolate)	Wellcome Trust Sanger Institute	^b^Traces-0CerOby (ERS007592)	*1069*	*6*	[2]
DT8	S81-848 (Veterinary isolate)	Institut Pasteur	Traces-0PArkjM	*1069*	*6*	Enterobase
DT8	MS150057 (Clinical isolate)	NSSLRL	Traces-0xVpmwI	*2260*	*708*	Enterobase
DT30	MS57 (Control DT30)	NSSLRL	^a^Traces-0aYyWix (ERS640854)	*812*	*250*	[8]
DT8	M12-2001 (Control DT8)	NSSLRL	^a^Traces-0Jyulvx (PRJEB18673)	*812*	*250*	This study
DT8	M15-2006 (Control DT8)	NSSLRL	^a^Traces-0WxCKWi (PRJEB18673)	*812*	*250*	This study
DT8	MS32 (Control DT8)	NSSLRL	^a^Traces-0dPdGho (PRJEB18673)	*812*	*250*	This study
*CRISPR/CRISPOL type among phage types DT104, DT104b and U302 of S.* Typhimurium						
DT104b	MS130531 (Control DT104b)	NSSLRL	^a^Traces-0ptnSId (PRJEB18673)	*12*	*21*	This study
U302	M18-2006 (Control U302)	NSSLRL	^a^Traces-0rRUdtU (PRJEB18673)	*12*	*21*	This study
DT104	TM75-339 (No data)	Institut Pasteur	Traces-0dpLsNp	*12*	*21*	Enterobase
DT104	MS150098 (Clinical isolate)	NSSLRL	Traces-0VNfjhC	*12*	*21*	Enterobase
DT104	MS150095 (Clinical isolate)	NSSLRL	Traces-0ohRXMQ	*12*	*21*	Enterobase
DT104b	MS150159 (Clinical isolate)	NSSLRL	Traces-0mdEaBO	*12*	*21*	Enterobase
DT104b	MS150253 (Clinical isolate)	NSSLRL	Traces-0VfHkWp	*7556*	*315*	Enterobase
DT104	MS150005 (Clinical isolate)	NSSLRL	Traces-0ehJIGG	*5000*	*168*	Enterobase
CRISPR/CRISPOL type among phage types DT99, DT56, U319 and DT40 of *S.* Typhimurium						
DT99	DT99 (Clinical isolate)	Wellcome Trust Sanger Institute	^b^Traces-0fQeupq (ERS007596)	*7433*	*14*	[2]
DT56	DT56 (Clinical isolate)	Wellcome Trust Sanger Institute	^b^Traces-0WirVGQ (ERS007588)	*7433*	*14*	[2]
U319	U319 (Clinical isolate)	Wellcome Trust Sanger Institute	^b^Traces-0nXusuL (ERS007613)	*7433*	*14*	[2]
DT40	S05-2864 (Clinical isolate)	Institut Pasteur	Traces-0PxGcXB	*7433*	*14*	Enterobase
DT40	M20-2006 (Control isolate)	NSSLRL	^a^Traces-0rGCwUc (PRJEB18673)	*9520*	*1637*	This study
DT40	M19-2003 (Control isolate)	NSSLRL	^a^Traces-0nxmoMB (PRJEB18673)	*9519*	*1636*	This study
DT40	CQ 40	Institut Pasteur	Traces-0LSHwEV	*745*	*18*	Enterobase
CRISPR/CRISPOL type among phage types DT120, DT7a, DT193 and untypable strains of *S.* Typhimurium						
DT120	S02-3776 (Clinical isolate)	Institut Pasteur	Traces-0yQDdlW	9921	1759	Enterobase
DT120	07_2198 (No Data)	Institut Pasteur	Traces-0pKTfCi	9911	1753	Enterobase
DT120	M16-2000 (Control DT120)	NSSLRL	^a^Traces-0fEcWgz (PRJEB18673)	9510	1428	Enterobase
DT7a	MS120840 (Control DT7a)	NSSLRL	^a^Traces-0psYyDm (PRJEB18673)	9510	1428	Enterobase
DT120	S/20160374 (Clinical isolate)	SSSCDRL	Traces-0CeRVgg	322	1	Enterobase
DT120	S/20160407 (Clinical isolate)	SSSCDRL	Traces-0agMeAc	322	1	Enterobase
DT20a	MS150110 (Clinical isolate)	NSSLRL	Traces-0isgxxB	322	1	Enterobase
Untypable	MS150097 (Clinical isolate)	NSSLRL	Traces-0VSlIab	322	1	Enterobase
DT193	MS150007 (Clinical isolate)	NSSLRL	Traces-0vpTyIh	322	1	Enterobase
DT193	MS150252 (Clinical isolate)	NSSLRL	Traces-0WAKQQZ	317	2	Enterobase
CRISPR/CRISPOL type among phage types DT12, DT3 and DT193a of *S.* Typhimurium						
DT12	DT12 (Clinical isolate)	Wellcome Trust Sanger Institute	^b^Traces-0kmZJki (ERS007564)	*5268*	*19*	[2]
DT12	S02-2651 (Clinical isolate)	Institut Pasteur	Traces-0FbQprS	*774*	*46*	Enterobase
DT3	S81-482 (Clinical isolate)	Institut Pasteur	Traces-0pUCktc	*5268*	*19*	Enterobase
DT3	S81-531 (Veterinary isolate)	Institut Pasteur	Traces-0pGWuNa	*539*	*13*	Enterobase
DT193a	MS120454 (Clinical isolate)	NSSLRL	Traces-0hfCzzz	*774*	*46*	Enterobase
CRISPR/CRISPOL type among phage types DT135, DT191a and RDNC strains of *S.* Typhimurium						
DT135	DT135 (Clinical isolate)	Wellcome Trust Sanger Institute	^b^Traces-0xEkcLV ERS007567	*5753*	*396*	[2]
DT135	MS150100 (Clinical isolate)	NSSLRL	Traces-0fqzVBN	*3247*	*66*	Enterobase
DT135	MS150112 (Clinical isolate)	NSSLRL	Traces-0TpmttL	*91*	*4*	Enterobase
DT135	MS150180 (Clinical isolate)	NSSLRL	Traces-0FksMUv	*91*	*4*	Enterobase
DT191a	DT191a (Clinical isolate)	Wellcome Trust Sanger Institute	^b^Traces-0KhAoGt ERS007574	*91*	*4*	[2]
RDNC	MS150102 (Clinical isolate)	NSSLRL	Traces-0bmnIRV	*91*	*4*	Enterobase
RDNC	MS150230 (Clinical isolate)	NSSLRL	Traces-0vTHNcg	*91*	*4*	Enterobase
RDNC	MS150009 (Clinical isolate)	NSSLRL	Traces-0Zipaoz	*9404*	*1614*	Enterobase

^a^Accession Numbers in Enterobase of control phage types of *S.* Typhimurium sequenced in this study. The Accession Number in ENA is also provided

^b^Accession Numbers in Enterobase of clinical isolates of *S.* Typhimurium used in this study. The Accession Number in ENA is also provided

#### In silico CRISPR and CRISPOL analysis

PE reads of different phage types of *S.* Typhimurium were also assembled using Enterobase (https://enterobase.warwick.ac.uk/) where CRISPRs and CRISPOL were called directly from the raw reads rather than the assembly.

Enterobase was used to determine the CRISPR type and CRISPOL type of all phage types of *S.* Typhimurium. In Enterobase, each phage type of *S.* Typhimurium was assigned unique accession number (Tables 1, 2).

Previously, sequenced CRISPR loci of different phage types of *S.* Typhimurium using polymerase chain reaction (PCR) [5] were also included in this study (Table 3).

**Table 3:** CRISPOL type among different phage types of *S.* Typhimurium

Phage type	Isolate ID (source)	Lab	Accession number CRISPR1 locus CRISPR2 locus		*CRISPOL type
DT104	02-1540 (Clinical isolate)	Institut Pasteur	JF724217	JF724959	*30*
DT104	05-2975 (Clinical isolate)	Institut Pasteur	JF724458	JF725631	*31*
DT104	02-8319 (Clinical isolate)	Institut Pasteur	JF724357	JF725099	*24*
DT104	02-4467 (Clinical isolate)	Institut Pasteur	JF724278	JF725020	*23*
DT104	02-4217 (Clinical isolate)	Institut Pasteur	JF724270	JF725012	*20*
DT104	02-3830 (Clinical isolate)	Institut Pasteur	JF724255	JF724997	*22*
DT104	02-3169 (Clinical isolate)	Institut Pasteur	JF724237	JF724979	*21*
DT120	02-5783 (Clinical isolate)	Institut Pasteur	JF724308	JF725050	*21*
DT120	02-4908 (Clinical isolate)	Institut Pasteur	JF724290	JF725032	*34*
U302	02-3709 (Clinical isolate)	Institut Pasteur	JF724252	JF724994	*21*
U302	02-5064 (Clinical isolate)	Institut Pasteur	JF724292	JF725034	*25*
DT2	81-506 (Veterinary isolate)	Institut Pasteur	JF724622	JF725354	*54*
DT2	01-1639 (Veterinary isolate)	Institut Pasteur	JF724170	JF724912	*55*
RDNC	81-748 (Clinical isolate)	Institut Pasteur	JF724624	JF725356	*33*
RDNC	DK19 (Clinical isolate)	Institut Pasteur	JF724652	JF725384	*12*
RDNC	07-4489 (Clinical isolate)	Institut Pasteur	JF724524	JF725256	*53*
DT1	02-0915 (Clinical isolate)	Institut Pasteur	JF724204	JF724946	*14*
DT40	05-2864 (Clinical isolate)	Institut Pasteur	JF724454	JF725196	*14*
DT1	81-481 (ND)	Institut Pasteur	JF724620	JF725352	*11*
DT74	DK24 (Clinical isolate)	Institut Pasteur	JF724648	JF725380	*11*
DT1	1000-7816-1 (Veterinary isolate)	Institut Pasteur	JF724578	JF725310	*46*
DT186	02-1015 (Clinical isolate)	Institut Pasteur	JF724205	JF724947	*46*
DT12	02-2651 (Clinical isolate)	Institut Pasteur	JF724232	JF724974	*46*
DT42	1000-7810-1 (Veterinary isolate)	Institut Pasteur	JF724577	JF725309	*46*
DT7	07-2537 (Clinical isolate)	Institut Pasteur	JF724521	JF725253	*1*
DT193	07-7741 (Clinical isolate)	Institut Pasteur	JF724531	JF725263	*1*
U311	07-8113 (Clinical isolate)	Institut Pasteur	JF724532	JF725264	*1*
DT41	07-5354 (Clinical isolate)	Institut Pasteur	JF724527	JF725259	*1*

CRISPR type was not determined as the whole genome sequence is not available for these strains

*CRISPOL type was determined by Fabre et al. [5]

PE reads of *S.* Typhimurium phage type DT8 associated with a foodborne outbreak in the summer of 2013 in the States of Jersey [10] were downloaded from ENA; study Accession Number PRJNA248792 (http://www.ebi.ac.uk/ena/data/view/PRJNA248792) and assembled by Enterobase. CRISPR and CRISPOL types were determined for all outbreak strains using Enterobase (Additional file 1: Table S1).

Spacers sequence within the assembled genomes of outbreak and non-outbreak associated DT8 strains were also characterized using CRISPRFinder (http://crispr.i2bc.paris-saclay.fr/Server/) (Additional file 1: Table S1).

## Results

In silico analysis of genome sequences of control and well documented phage types of *S.* Typhimurium revealed two CRISPR loci, CRISPR-1 and CRISPR-2, within all phage types of *S.* Typhimurium. Although DRs are almost identical among all phage types of *S.* Typhimurium spacers sequences within the CRISPR loci are not unique to the phage type as strains belong to the same phage type have different spacers and subsequently different CRISPR/CRISPOL type (Table 1) furthermore, different phage types have identical spacers and same CRISPR/CRISPOL type (Table 2).

### Different CRISPR/CRISPOL type within the same phage type of *S.* Typhimurium

In Table 1, three strains of *S.* Typhimurium that belong to phage type DT1 including strains DT1, TM 68-619 and TM 65-111 have different spacers and subsequently show different CRISPR/CRISPOL type; 8579/430, 2536/54 and 7387/90 respectively. Two strains belong to phage type DT10 have different CRISPR/CRISPOL type; MS34 (9509/1629) and S81-784 (9913/1688). Two strains belong to phage type DT15a have different CRISPR/CRISPOL type; 9517/1634 in isolate MS41 and 9916/1756 in isolate S81-798. Moreover, three strains belong to DT41 have different CRISPR/CRISPOL type; 9513/1630 in isolate M11-2004, 7434/223 in isolate CQ 41 and 9929/1766 in isolate S02-0321.

### Identical CRISPR/CRISPOL type within different phage types of *S.* Typhimurium

#### CRISPR/CRISPOL type among phage types DT8 and DT30

Identical spacers were detected among different phage types of *S.* Typhimurium. For example, three strains of DT8 including M12-2001, M15-2006 and MS32 have the same CRISPR/CRISPOL type (812/250) as a strain belongs to phage type DT30 (MS57). Moreover, different strains belong to phage type DT8 have different CRISPR/CRISPOL type; M18-2003 (1069/6) and MS150057 (2260/708) (Table 2).

Interestingly, *S.* Typhimurium DT8 strains associated with the foodborne outbreak in the summer of 2013 in the States of Jersey [10] showed identical CRISPR/CRISPOL type (1069/6) however, the same CRISPR/CRISPOL type were reported in other DT8 strains that do not belong to the outbreak as confirmed by WGS [10]. Identical spacers were detected among outbreak associated and non-outbreak associated DT8 strains (Additional file 1: Table S1).

#### CRISPR/CRISPOL type among phage types DT104, DT104b and U302

Variations in the CRISPR/CRISPOL type among strains of the same phage type such as DT104 and DT104b have been also noticed (Table 2). Although three strains of *S.* Typhimurium phage type DT104 including TM75-339, MS150098 and MS150095, have identical spacer sequences and CRISPR/CRISPOL type (12/21) the same CRISPR/CRISPOL type is present in different phage types including U302 (M18-2006; 12/21) and DT104b (MS130531; 12/21).

#### CRISPR/CRISPOL type among phage types DT40, DT56, DT99 and U319

Strains of *S.* Typhimurium belong to different phage types such as DT99, DT56, U319 and DT40 (S05-2864) have identical spacer sequences and identical CRISPR/CRISPOL type (7433/14). Moreover, several strains belong to phage type DT40 including S05-2864, M20-2006, M19-2003 and CQ 40 have different CRISPR/CRISPOL type; 7433/14, 9520/1637, 9519/1636 and 745/18 respectively (Table 2).

#### CRISPR/CRISPOL type among phage types DT7a, DT20a, DT120, DT193 and untypable strains

In Table 2, strains of *S.* Typhimurium belong to phage type DT120 have different spacers and subsequently different CRISPR/CRISPOL type including S02-3776 (9921/1759), 07_2198 (9911/1753), M16-2000 (9510/1428), and S/20160374 (322/1).

Interestingly, a strains of phage type DT120 (M16-2000) has identical spacers and CRISPR/CRISPOL type (9510/1428) as another strain belongs to phage type DT7a (MS120840). Moreover, some strains belong to phage types DT120 (S/20160374 and S/20160407), DT20a (MS150110), DT193 (MS150007) and untypable strain (MS150097) have identical spacers and therefore share the same CRISPR/CRISPOL type (322/1). Different strains belong to phage type DT193 have different spacers and CRISPR/CRISPOL type; MS150007 (322/1) and MS150252 (317/2).

#### CRISPR/CRISPOL type among phage types DT3, DT12 and DT193a

Some strains of phage types DT12 (DT12) and DT3 (S81-482) have identical spacers and identical CRISPR/CRISPOL type; 5268/19. Moreover, a strain belongs to DT12 (S02-2651) has identical CRISPR/CRISPOL type, 774/46, as a strain belongs to phage type DT193a (MS120454) (Table 2).

#### CRISPR/CRISPOL type among phage types DT135, DT191a and RDNC

Identical spacer sequences and CRISPR/CRISPOL type (91/4) were detected in different phage types of *S.* Typhimurium including DT135 (MS150112 and MS150180), DT191a (DT19a) and strains that react with phages but do not confirm to recognized pattern (RDNC) (MS150102 and MS150230). Furthermore, other strains belong to phage type DT135 show different spacers and subsequently different CRISPR/CRISPOL type; 5753/396 in DT135 and 3247/66 in MS150100 (Table 2).

### CRISPOL assay confirms the no relation among phage type and CRISPRs

CRISPOL assay developed by Fabre et al. [5] when carried out on representative phage types of *S.* Typhimurium it reveals that there is no relation among the phage type and the CRISPOL type as strains belong to the same phage type have different CRISPOL type as seen in DT104 strains (Table 3). On the other hand, different phage types including DT7, DT193, U311, DT41 showed identical CRISPOL type as ‘1’ (Table 3).

## Discussion

*Salmonella* Typhimurium is the most dominant *Salmonella* serovar around the world and has been associated with foodborne outbreaks in both developing and high-income countries [1, 11] and infection can result in bacteraemia and invasive disease [12, 13]. Epidemiological characterization of *S.* Typhimurium is therefore very crucial for the surveillance and outbreak investigation.

Phage typing system [3] has been a very useful phenotypical, definitive method for epidemiological characterization of *S.* Typhimurium and identification of the source of infection [14–17]. Although it has been suggested that the high throughput CRISPR typing and subtyping have the potential to replace traditional phage typing [5] this study demonstrates that It is impossible for CRISPR typing and CRISPOl assay to replace phage typing for epidemiological characterization of *S.* Typhimurium as there is no correlation between the phage type and the CRISPR/CRISPOL type.

Interestingly, *S.* Typhimurium DT8 strains associated with the foodborne outbreak in the summer of 2013 in the States of Jersey [10] showed identical CRISPR/CRISPOL type however, the same CRISPR/CRISPOL type were reported in other DT8 strains that do not belong to the outbreak as confirmed by WGS [10]. Detection of identical spacers among outbreak associated and non-outbreak associated DT8 strains reveals the limitation of CRISPR typing and subtyping in investigation of outbreaks.

The MDR DT104 strain of *S.* Typhimurium has been associated with foodborne outbreaks all over the world and phage typing was very successful in epidemiological characterization of the outbreak and identification of the source [18–20] however in this study strains belong to DT104 showed different spacers and subsequently different CRISPR/CRISPOL type therefore CRISPR typing and CRISPOL assay cannot be used in public health laboratories to determine the epidemiological relation among *S.* Typhimurium isolates.

The presence of CRISPR/CRISPOL type within the same phage type and the presence of identical spacers among different phage types of *S.* Typhimurium confirms the limitations of CRISPR typing and subtyping for the epidemiological surveillance and outbreak investigation of *S.* Typhimurium.

There is no doubt that rapid WGS will shape the future of diagnostic microbiology as it has the potential to replace the routine typing and subtyping methods including Anderson phage typing system for the surveillance of outbreaks caused by different *Salmonella* serovars in real-time [10, 21, 22]. However, in the meantime, traditional phage typing scheme of *S.* Typhimurium remains the gold standard method for subtyping of *S.* Typhimurium for laboratory surveillance and outbreak investigation despite its technical limitations. Furthermore, it represents an ideal model for studying the complex dynamics of phage-host interaction [8].

In conclusion, high throughput CRISPR/CRISPOL typing might be useful for the discrimination among different *Salmonella* serovars however it is not useful for the epidemiological surveillance and outbreak investigation of *S.* Typhimurium and phage typing, until it is replaced by WGS, is still the gold standard method for epidemiological surveillance of *S.* Typhimurium.

## Limitations

More outbreaks of *S.* Typhimurium caused by phage types other than DT8 can be included to confirm the unsuitability of CRISPR typing in epidemiological surveillance and outbreak investigation of *S.* Typhimurium.

## Additional file

**Additional file 1: Table S1.** CRISPR and CRISPOL types of outbreak and non-outbreak associated DT8 strains of *S.* Typhimurium. Identical CRISPR and CRISPOL types were detected among outbreak and non-outbreak strains.

## Abbreviations

CRISPR: clustered regular interspaced short palindromic repeats
DT: phage type
MDR: multidrug resistant
NSSLRL: National Salmonella Shigella Listeria Reference Laboratory
NTS: nontyphoidal salmonella
PE: paired end
PCR: polymerase chain reaction
RDNC: strains that react with phages but do not confirm to recognized pattern
SSSCDRL: Scottish Salmonella, Shigella and Clostridium difficile Reference Laboratory
*S.* Typhimurium: *Salmonella* Typhimurium
WGS: whole genome sequencing
