# Supplementary material for: Phage typing or CRISPR typing for epidemiological surveillance of Salmonella Typhimurium?
Source: BMC Res Notes. 2017 Nov 7;10:578. doi: 10.1186/s13104-017-2878-0 (PMC5678594; doi:10.1186/s13104-017-2878-0)
Supplement: Supplementary file 1 — Additional file 1: Table S1. CRISPR and CRISPOL types of outbreak and non-outbreak associated DT8 strains of S. Typhimurium. Identical CRISPR and CRISPOL types were detected among outbreak and non-outbreak strains. [file 13104_2017_2878_MOESM1_ESM.docx]

**Table S1: CRISPR and CRISPOL types of outbreak and non-outbreak associated DT8 strains of *S.* Typhimurium**

| **Strain ID** | **ENA Accession number** | **Receipt date** | **Source** | **Enterobase Accession number** | **CRISPR type** | **CRISPOL type** |
| --- | --- | --- | --- | --- | --- | --- |
| **OUTBREAK ASSOSIATED DT8 strains of *S.* Typhimurium** | | | | | | |
| H133000654 | SRR2163487 | 22/07/2013 | Human | traces-0yFVtEJ | 1069 | 6 |
| H133060375 | SRR2163444 | 25/07/2013 | Mayonnaise | traces-0jBkjNE | 1069 | 6 |
| H13306376 | SRR2163445 | 25/07/2013 | Mayonnaise | traces-0dGjRLW | 1069 | 6 |
| H133060377 | SRR2163446 | 25/07/2013 | Mayonnaise | traces-0OUwaTM | 1069 | 6 |
| H133060378 | SRR2163488 | 25/07/2013 | Mayonnaise | traces-0DBiaaH | 1069 | 6 |
| H132940743 | SRR2163469 | 17/07/2013 | Human | traces-0KhTEXU | 1069 | 6 |
| H132940744 | SRR2163437 | 17/07/2013 | Human | traces-0IPDGvh | 1069 | 6 |
| H132940745 | SRR2163440 | 17/07/2013 | Human | traces-0VRsUCN | 1069 | 6 |
| H132940746 | SRR2163471 | 17/07/2013 | Human | traces-0FueXxu | 1069 | 6 |
| H132940748 | SRR2163458 | 17/07/2013 | Human | traces-0VQoRAI | 1069 | 6 |
| H132940749 | SRR2163465 | 17/07/2013 | Human | traces-0lXtqHW | 1069 | 6 |
| H132940750 | SRR2163442 | 17/07/2013 | Human | traces-0dtaLTL | 1069 | 6 |
| H132940751 | SRR2163436 | 17/07/2013 | Human | traces-0peCZhu | 1069 | 6 |
| H132940753 | SRR2163434 | 17/07/2013 | Human | traces-0jsnddC | 1069 | 6 |
| H132940754 | SRR2163459 | 17/07/2013 | Human | traces-0oesFol | 1069 | 6 |
| H132940756 | SRR2163484 | 17/07/2013 | Human | traces-0cXyjGF | 1069 | 6 |
| H133000645 | SRR2163480 | 22/07/2013 | Human | traces-0MptDPD | 1069 | 6 |
| H133300609 | SRR2163438 | 12/08/203 | Human | traces-0TWepNb | 1069 | 6 |
| H132300541 | SRR2163435 | 03/06/2013 | Human | traces-0HQZVLf | 1069 | 6 |
| **NON-OUTBREAK ASSOSIATED DT8 strains of *S.* Typhimurium** | | | | | | |
| H133260293 | SRR2163486 | 08/08/2013 | Human | traces-0CoTGFn | 1069 | 6 |
| H132780266 | SRR2163433 | 05/07/2013 | Human | traces-0uLjdHA | 1069 | 6 |
| H132960590 | SRR2163456 | 18/07/2013 | Human | traces-0QbJMrF | 1069 | 6 |
| H132920685 | SRR2163474 | 16/07/2013 | Human | traces-0YYssib | 1069 | 6 |
| H132980531 | SRR2163489 | 19/07/2013 | Human | traces-0SzSijx | 1069 | 6 |
| H121600325 | SRR1635127 | 16/04/2012 | Human | traces-0hhCVRQ | 1069 | 6 |
| H122720573 | SRR1645461 | 03/07/2012 | Human | traces-0VxyMld | 1069 | 6 |
| H12320661 | SRR1645554 | 25/09/2012 | Human | traces-0UJrUip | 1069 | 6 |
| H123020544 | SRR1645246 | 24/07/2012 | Human | traces-0VJgsMn | 1069 | 6 |
| H132020501 | Srr2163450 | 14/05/2013 | Human | traces-0sMnwms | 1069 | 6 |
| H122020454 | SRR1646143 | 15/05/2012 | Human | traces-0oVfhNe | 1069 | 6 |
| H124860455 | SRR1645387 | 29/11/2012 | Human | traces-0gFaInb | 1069 | 6 |
| H133040470 | SRR2163462 | 24/07/2013 | Human | traces-0JTAKFm | 1069 | 6 |
| H1330400611 | SRR2163457 | 19/08/2013 | Human | traces-0ghtsQf | 1069 | 6 |

**CRISPR type for all outbreak and non-outbreak associated DT8 strains of *S.* Typhimurium as determined by Enterobase: (1069)**

**CRISPR1:** DR22-STM1-DR-STM2-DR14-STM3-DR-STM25-DR-STM26-DR-STM27-DR-STM4-DR-STM5-DR-STM6-DR-STM7-DR-STM8-DR-STM9-DR-STM10-DR-STM11-DR-STM12var1-DR-STM28-DR-STM13-DR28-STM14-DR-STM15-DR-STM16-DR-STM19-DR-STM20-DR-STM24-DR)

**CRISPR2:** DR1C-STMB0-DR-STMB32-DR8-STMB1-DR-STMB2-DR-STMB3-DR-STMB5-DR13-STMB6-DR4-STMB30-DR-STMB31-DR

**CRISPOL type for all outbreak and non-outbreak associated DT8 strains of *S.* Typhimurium as determined by Entrobase:** (**6)**

Spacers: STM01-STM02-STM03-STM25-STM26-STM27-STM04-STM05-STM06-STM07-STM08-STM09-STM10-STM11-STM12var1-STM28-STM13-STM14-STM15-STM16-STM19-STM20-STM24-STMB0-STMB32-STMB1-STMB2-STMB3-STMB5-STMB6-STMB30-STMB31

**Spacers within outbreak and non-outbreak DT8 strains of Salmonella Typhimurium as determined by CRISPRFinder**

**H121600325**

>spacer1

TTTGCCGATCCCCTTCCAGACCACCCTTTACA

>spacer2

TGCAGAGTTGTATCTTACCCTGTTTACGTTTC

>spacer3

CGTCACTTTCTGACATTTTATTCAGTTCGTTA

>spacer4

TCATTTCTGGACGGGGCTGTGTGACGAATACG

>spacer5

GGATATGTGAAGTTCAGGTAGCCCATTACGCA

>spacer6

TTGATCGAGAGTGCGAAGAGGCAGAACGGGCA

>spacer7

CAGGTTATGCGCAAAAATTAATTCATATTATA

>spacer8

GACGAGTTCTGGAAATGGTTAGCTGATAAAGA

>spacer9

CGTTCATCGGCAGCGTCACGCAATATGAAGAT

>spacer1

TGAGCAACGACAGTAAATAATTTTTCGTGCTG

>spacer2

CCAGTGGGCGTAGCCAGCTCATCGCTATTTTGC

>spacer3

CGTTGCGGATTATCGTTAAGACTGAAGGAAGT

>spacer4

TTGCAGGGCGATATTGTTGTTGGTGAATGGGA

>spacer5

CGTCGCGGAAAATTTCGCATTGACGATAAAGA

>spacer6

TTACGTGTTTATTCATCTGTTGCATTAGATTC

>spacer7

GAGGCGTACAGGCTGTTAGATGAGAAATTACC

>spacer8

ACGCCCCGAATGTGTTTGCCTCGCCCGCTGCC

>spacer9

TGGATTATCTGTATCTTACGGAAGTGGGCGCG

>spacer10

GTCGTTCATCAGGCACTACCGGCACTTTCTGG

>spacer11

ATATTCGCCGCTTTCCATTTACCGAACGTAAC

>spacer12

CCACGTTCGGCGATGTTGGCCCCATCGGTCCA

>spacer13

AAACGGTAGTGTTTTAAAACCGTTTCGAGGTGC

>spacer15

AGCCGTTTCCGCTAAATACCCCCGCAGTGATT

>spacer16

TTCTTGAATATGATTGCGGGTATATGTGGATA

>spacer17

TCTGGTTATAACATCGCAGCAAAATCAAAAGA

>spacer18

GCACTATTTCGAATGTCTCGACGCCAGATTTA

>spacer19

AACGAATTGAGACTATTAGAGATTATTCGCCT

>spacer20

GCAACCCATTAATTAACTAAGCAGTAATAAAC

>spacer21

TGACGAGGTGCGAGCGATGGTATCAAGGCCTA

>spacer22

GGTTAACCAGGGGTTTTTCCCCACTATTTCGC

>spacer23

AGGGGCGTTCCGCAGTCGACAAGGGCTGAAAA

**H122020454**

>spacer1

TTTGCCGATCCCCTTCCAGACCACCCTTTACA

>spacer2

TGCAGAGTTGTATCTTACCCTGTTTACGTTTC

>spacer3

CGTCACTTTCTGACATTTTATTCAGTTCGTTA

>spacer4

TCATTTCTGGACGGGGCTGTGTGACGAATACG

>spacer5

GGATATGTGAAGTTCAGGTAGCCCATTACGCA

>spacer6

TTGATCGAGAGTGCGAAGAGGCAGAACGGGCA

>spacer7

CAGGTTATGCGCAAAAATTAATTCATATTATA

>spacer8

GACGAGTTCTGGAAATGGTTAGCTGATAAAGA

>spacer9

CGTTCATCGGCAGCGTCACGCAATATGAAGAT

>spacer1

TGAGCAACGACAGTAAATAATTTTTCGTGCTG

>spacer2

CCAGTGGGCGTAGCCAGCTCATCGCTATTTTGC

>spacer3

CGTTGCGGATTATCGTTAAGACTGAAGGAAGT

>spacer4

TTGCAGGGCGATATTGTTGTTGGTGAATGGGA

>spacer5

CGTCGCGGAAAATTTCGCATTGACGATAAAGA

>spacer6

TTACGTGTTTATTCATCTGTTGCATTAGATTC

>spacer7

GAGGCGTACAGGCTGTTAGATGAGAAATTACC

>spacer8

ACGCCCCGAATGTGTTTGCCTCGCCCGCTGCC

>spacer9

TGGATTATCTGTATCTTACGGAAGTGGGCGCG

>spacer10

GTCGTTCATCAGGCACTACCGGCACTTTCTGG

>spacer11

ATATTCGCCGCTTTCCATTTACCGAACGTAAC

>spacer12

CCACGTTCGGCGATGTTGGCCCCATCGGTCCA

>spacer13

AAACGGTAGTGTTTTAAAACCGTTTCGAGGTGC

>spacer15

AGCCGTTTCCGCTAAATACCCCCGCAGTGATT

>spacer16

TTCTTGAATATGATTGCGGGTATATGTGGATA

>spacer17

TCTGGTTATAACATCGCAGCAAAATCAAAAGA

>spacer18

GCACTATTTCGAATGTCTCGACGCCAGATTTA

>spacer19

AACGAATTGAGACTATTAGAGATTATTCGCCT

>spacer20

GCAACCCATTAATTAACTAAGCAGTAATAAAC

>spacer21

TGACGAGGTGCGAGCGATGGTATCAAGGCCTA

>spacer22

GGTTAACCAGGGGTTTTTCCCCACTATTTCGC

>spacer23

AGGGGCGTTCCGCAGTCGACAAGGGCTGAAAA

**H122720573**

>spacer1

TTTGCCGATCCCCTTCCAGACCACCCTTTACA

>spacer2

TGCAGAGTTGTATCTTACCCTGTTTACGTTTC

>spacer3

CGTCACTTTCTGACATTTTATTCAGTTCGTTA

>spacer4

TCATTTCTGGACGGGGCTGTGTGACGAATACG

>spacer5

GGATATGTGAAGTTCAGGTAGCCCATTACGCA

>spacer6

TTGATCGAGAGTGCGAAGAGGCAGAACGGGCA

>spacer7

CAGGTTATGCGCAAAAATTAATTCATATTATA

>spacer8

GACGAGTTCTGGAAATGGTTAGCTGATAAAGA

>spacer9

CGTTCATCGGCAGCGTCACGCAATATGAAGAT

>spacer1

TGAGCAACGACAGTAAATAATTTTTCGTGCTG

>spacer2

CCAGTGGGCGTAGCCAGCTCATCGCTATTTTGC

>spacer3

CGTTGCGGATTATCGTTAAGACTGAAGGAAGT

>spacer4

TTGCAGGGCGATATTGTTGTTGGTGAATGGGA

>spacer5

CGTCGCGGAAAATTTCGCATTGACGATAAAGA

>spacer6

TTACGTGTTTATTCATCTGTTGCATTAGATTC

>spacer7

GAGGCGTACAGGCTGTTAGATGAGAAATTACC

>spacer8

ACGCCCCGAATGTGTTTGCCTCGCCCGCTGCC

>spacer9

TGGATTATCTGTATCTTACGGAAGTGGGCGCG

>spacer10

GTCGTTCATCAGGCACTACCGGCACTTTCTGG

>spacer11

ATATTCGCCGCTTTCCATTTACCGAACGTAAC

>spacer12

CCACGTTCGGCGATGTTGGCCCCATCGGTCCA

>spacer13

AAACGGTAGTGTTTTAAAACCGTTTCGAGGTGC

>spacer15

AGCCGTTTCCGCTAAATACCCCCGCAGTGATT

>spacer16

TTCTTGAATATGATTGCGGGTATATGTGGATA

>spacer17

TCTGGTTATAACATCGCAGCAAAATCAAAAGA

>spacer18

GCACTATTTCGAATGTCTCGACGCCAGATTTA

>spacer19

AACGAATTGAGACTATTAGAGATTATTCGCCT

>spacer20

GCAACCCATTAATTAACTAAGCAGTAATAAAC

>spacer21

TGACGAGGTGCGAGCGATGGTATCAAGGCCTA

>spacer22

GGTTAACCAGGGGTTTTTCCCCACTATTTCGC

>spacer23

AGGGGCGTTCCGCAGTCGACAAGGGCTGAAAA

H123020544

>spacer1

TTTTCAGCCCTTGTCGACTGCGGAACGCCCCT

>spacer2

GCGAAATAGTGGGGAAAAACCCCTGGTTAACC

>spacer3

TAGGCCTTGATACCATCGCTCGCACCTCGTCA

>spacer4

GTTTATTACTGCTTAGTTAATTAATGGGTTGC

>spacer5

AGGCGAATAATCTCTAATAGTCTCAATTCGTT

>spacer6

TAAATCTGGCGTCGAGACATTCGAAATAGTGC

>spacer7

TCTTTTGATTTTGCTGCGATGTTATAACCAGA

>spacer8

TATCCACATATACCCGCAATCATATTCAAGAA

>spacer9

AATCACTGCGGGGGTATTTAGCGGAAACGGCT

>spacer11

GCACCTCGAAACGGTTTTAAAACACTACCGTTT

>spacer12

TGGACCGATGGGGCCAACATCGCCGAACGTGG

>spacer13

GTTACGTTCGGTAAATGGAAAGCGGCGAATAT

>spacer14

CCAGAAAGTGCCGGTAGTGCCTGATGAACGAC

>spacer15

CGCGCCCACTTCCGTAAGATACAGATAATCCA

>spacer16

GGCAGCGGGCGAGGCAAACACATTCGGGGCGT

>spacer17

GGTAATTTCTCATCTAACAGCCTGTACGCCTC

>spacer18

GAATCTAATGCAACAGATGAATAAACACGTAA

>spacer19

TCTTTATCGTCAATGCGAAATTTTCCGCGACG

>spacer20

TCCCATTCACCAACAACAATATCGCCCTGCAA

>spacer21

ACTTCCTTCAGTCTTAACGATAATCCGCAACG

>spacer22

GCAAAATAGCGATGAGCTGGCTACGCCCACTGG

>spacer23

CAGCACGAAAAATTATTTACTGTCGTTGCTCA

>spacer1

ATCTTCATATTGCGTGACGCTGCCGATGAACG

>spacer2

TCTTTATCAGCTAACCATTTCCAGAACTCGTC

>spacer3

TATAATATGAATTAATTTTTGCGCATAACCTG

>spacer4

TGCCCGTTCTGCCTCTTCGCACTCTCGATCAA

>spacer5

TGCGTAATGGGCTACCTGAACTTCACATATCC

>spacer6

CGTATTCGTCACACAGCCCCGTCCAGAAATGA

>spacer7

TAACGAACTGAATAAAATGTCAGAAAGTGACG

>spacer8

GAAACGTAAACAGGGTAAGATACAACTCTGCA

>spacer9

TGTAAAGGGTGGTCTGGAAGGGGATCGGCAAA

H123920661

>spacer1

TTTGCCGATCCCCTTCCAGACCACCCTTTACA

>spacer2

TGCAGAGTTGTATCTTACCCTGTTTACGTTTC

>spacer3

CGTCACTTTCTGACATTTTATTCAGTTCGTTA

>spacer4

TCATTTCTGGACGGGGCTGTGTGACGAATACG

>spacer5

GGATATGTGAAGTTCAGGTAGCCCATTACGCA

>spacer6

TTGATCGAGAGTGCGAAGAGGCAGAACGGGCA

>spacer7

CAGGTTATGCGCAAAAATTAATTCATATTATA

>spacer8

GACGAGTTCTGGAAATGGTTAGCTGATAAAGA

>spacer9

CGTTCATCGGCAGCGTCACGCAATATGAAGAT

>spacer1

TGAGCAACGACAGTAAATAATTTTTCGTGCTG

>spacer2

CCAGTGGGCGTAGCCAGCTCATCGCTATTTTGC

>spacer3

CGTTGCGGATTATCGTTAAGACTGAAGGAAGT

>spacer4

TTGCAGGGCGATATTGTTGTTGGTGAATGGGA

>spacer5

CGTCGCGGAAAATTTCGCATTGACGATAAAGA

>spacer6

TTACGTGTTTATTCATCTGTTGCATTAGATTC

>spacer7

GAGGCGTACAGGCTGTTAGATGAGAAATTACC

>spacer8

ACGCCCCGAATGTGTTTGCCTCGCCCGCTGCC

>spacer9

TGGATTATCTGTATCTTACGGAAGTGGGCGCG

>spacer10

GTCGTTCATCAGGCACTACCGGCACTTTCTGG

>spacer11

ATATTCGCCGCTTTCCATTTACCGAACGTAAC

>spacer12

CCACGTTCGGCGATGTTGGCCCCATCGGTCCA

>spacer13

AAACGGTAGTGTTTTAAAACCGTTTCGAGGTGC

>spacer15

AGCCGTTTCCGCTAAATACCCCCGCAGTGATT

>spacer16

TTCTTGAATATGATTGCGGGTATATGTGGATA

>spacer17

TCTGGTTATAACATCGCAGCAAAATCAAAAGA

>spacer18

GCACTATTTCGAATGTCTCGACGCCAGATTTA

>spacer19

AACGAATTGAGACTATTAGAGATTATTCGCCT

>spacer20

GCAACCCATTAATTAACTAAGCAGTAATAAAC

>spacer21

TGACGAGGTGCGAGCGATGGTATCAAGGCCTA

>spacer22

GGTTAACCAGGGGTTTTTCCCCACTATTTCGC

>spacer23

AGGGGCGTTCCGCAGTCGACAAGGGCTGAAAA

**H124860455**

>spacer1

TTTTCAGCCCTTGTCGACTGCGGAACGCCCCT

>spacer2

GCGAAATAGTGGGGAAAAACCCCTGGTTAACC

>spacer3

TAGGCCTTGATACCATCGCTCGCACCTCGTCA

>spacer4

GTTTATTACTGCTTAGTTAATTAATGGGTTGC

>spacer5

AGGCGAATAATCTCTAATAGTCTCAATTCGTT

>spacer6

TAAATCTGGCGTCGAGACATTCGAAATAGTGC

>spacer7

TCTTTTGATTTTGCTGCGATGTTATAACCAGA

>spacer8

TATCCACATATACCCGCAATCATATTCAAGAA

>spacer9

AATCACTGCGGGGGTATTTAGCGGAAACGGCT

>spacer11

GCACCTCGAAACGGTTTTAAAACACTACCGTTT

>spacer12

TGGACCGATGGGGCCAACATCGCCGAACGTGG

>spacer13

GTTACGTTCGGTAAATGGAAAGCGGCGAATAT

>spacer14

CCAGAAAGTGCCGGTAGTGCCTGATGAACGAC

>spacer15

CGCGCCCACTTCCGTAAGATACAGATAATCCA

>spacer16

GGCAGCGGGCGAGGCAAACACATTCGGGGCGT

>spacer17

GGTAATTTCTCATCTAACAGCCTGTACGCCTC

>spacer18

GAATCTAATGCAACAGATGAATAAACACGTAA

>spacer19

TCTTTATCGTCAATGCGAAATTTTCCGCGACG

>spacer20

TCCCATTCACCAACAACAATATCGCCCTGCAA

>spacer21

ACTTCCTTCAGTCTTAACGATAATCCGCAACG

>spacer22

GCAAAATAGCGATGAGCTGGCTACGCCCACTGG

>spacer23

CAGCACGAAAAATTATTTACTGTCGTTGCTCA

>spacer1

ATCTTCATATTGCGTGACGCTGCCGATGAACG

>spacer2

TCTTTATCAGCTAACCATTTCCAGAACTCGTC

>spacer3

TATAATATGAATTAATTTTTGCGCATAACCTG

>spacer4

TGCCCGTTCTGCCTCTTCGCACTCTCGATCAA

>spacer5

TGCGTAATGGGCTACCTGAACTTCACATATCC

>spacer6

CGTATTCGTCACACAGCCCCGTCCAGAAATGA

>spacer7

TAACGAACTGAATAAAATGTCAGAAAGTGACG

>spacer8

GAAACGTAAACAGGGTAAGATACAACTCTGCA

>spacer9

TGTAAAGGGTGGTCTGGAAGGGGATCGGCAAA

H124860455

>spacer1

TTTTCAGCCCTTGTCGACTGCGGAACGCCCCT

>spacer2

GCGAAATAGTGGGGAAAAACCCCTGGTTAACC

>spacer3

TAGGCCTTGATACCATCGCTCGCACCTCGTCA

>spacer4

GTTTATTACTGCTTAGTTAATTAATGGGTTGC

>spacer5

AGGCGAATAATCTCTAATAGTCTCAATTCGTT

>spacer6

TAAATCTGGCGTCGAGACATTCGAAATAGTGC

>spacer7

TCTTTTGATTTTGCTGCGATGTTATAACCAGA

>spacer8

TATCCACATATACCCGCAATCATATTCAAGAA

>spacer9

AATCACTGCGGGGGTATTTAGCGGAAACGGCT

>spacer11

GCACCTCGAAACGGTTTTAAAACACTACCGTTT

>spacer12

TGGACCGATGGGGCCAACATCGCCGAACGTGG

>spacer13

GTTACGTTCGGTAAATGGAAAGCGGCGAATAT

>spacer14

CCAGAAAGTGCCGGTAGTGCCTGATGAACGAC

>spacer15

CGCGCCCACTTCCGTAAGATACAGATAATCCA

>spacer16

GGCAGCGGGCGAGGCAAACACATTCGGGGCGT

>spacer17

GGTAATTTCTCATCTAACAGCCTGTACGCCTC

>spacer18

GAATCTAATGCAACAGATGAATAAACACGTAA

>spacer19

TCTTTATCGTCAATGCGAAATTTTCCGCGACG

>spacer20

TCCCATTCACCAACAACAATATCGCCCTGCAA

>spacer21

ACTTCCTTCAGTCTTAACGATAATCCGCAACG

>spacer22

GCAAAATAGCGATGAGCTGGCTACGCCCACTGG

>spacer23

CAGCACGAAAAATTATTTACTGTCGTTGCTCA

>spacer1

ATCTTCATATTGCGTGACGCTGCCGATGAACG

>spacer2

TCTTTATCAGCTAACCATTTCCAGAACTCGTC

>spacer3

TATAATATGAATTAATTTTTGCGCATAACCTG

>spacer4

TGCCCGTTCTGCCTCTTCGCACTCTCGATCAA

>spacer5

TGCGTAATGGGCTACCTGAACTTCACATATCC

>spacer6

CGTATTCGTCACACAGCCCCGTCCAGAAATGA

>spacer7

TAACGAACTGAATAAAATGTCAGAAAGTGACG

>spacer8

GAAACGTAAACAGGGTAAGATACAACTCTGCA

>spacer9

TGTAAAGGGTGGTCTGGAAGGGGATCGGCAAA

**H132300541**

>spacer1

TTTGCCGATCCCCTTCCAGACCACCCTTTACA

>spacer2

TGCAGAGTTGTATCTTACCCTGTTTACGTTTC

>spacer3

CGTCACTTTCTGACATTTTATTCAGTTCGTTA

>spacer4

TCATTTCTGGACGGGGCTGTGTGACGAATACG

>spacer5

GGATATGTGAAGTTCAGGTAGCCCATTACGCA

>spacer6

TTGATCGAGAGTGCGAAGAGGCAGAACGGGCA

>spacer7

CAGGTTATGCGCAAAAATTAATTCATATTATA

>spacer8

GACGAGTTCTGGAAATGGTTAGCTGATAAAGA

>spacer9

CGTTCATCGGCAGCGTCACGCAATATGAAGAT

>spacer1

TGAGCAACGACAGTAAATAATTTTTCGTGCTG

>spacer2

CCAGTGGGCGTAGCCAGCTCATCGCTATTTTGC

>spacer3

CGTTGCGGATTATCGTTAAGACTGAAGGAAGT

>spacer4

TTGCAGGGCGATATTGTTGTTGGTGAATGGGA

>spacer5

CGTCGCGGAAAATTTCGCATTGACGATAAAGA

>spacer6

TTACGTGTTTATTCATCTGTTGCATTAGATTC

>spacer7

GAGGCGTACAGGCTGTTAGATGAGAAATTACC

>spacer8

ACGCCCCGAATGTGTTTGCCTCGCCCGCTGCC

>spacer9

TGGATTATCTGTATCTTACGGAAGTGGGCGCG

>spacer10

GTCGTTCATCAGGCACTACCGGCACTTTCTGG

>spacer11

ATATTCGCCGCTTTCCATTTACCGAACGTAAC

>spacer12

CCACGTTCGGCGATGTTGGCCCCATCGGTCCA

>spacer13

AAACGGTAGTGTTTTAAAACCGTTTCGAGGTGC

>spacer15

AGCCGTTTCCGCTAAATACCCCCGCAGTGATT

>spacer16

TTCTTGAATATGATTGCGGGTATATGTGGATA

>spacer17

TCTGGTTATAACATCGCAGCAAAATCAAAAGA

>spacer18

GCACTATTTCGAATGTCTCGACGCCAGATTTA

>spacer19

AACGAATTGAGACTATTAGAGATTATTCGCCT

>spacer20

GCAACCCATTAATTAACTAAGCAGTAATAAAC

>spacer21

TGACGAGGTGCGAGCGATGGTATCAAGGCCTA

>spacer22

GGTTAACCAGGGGTTTTTCCCCACTATTTCGC

>spacer23

AGGGGCGTTCCGCAGTCGACAAGGGCTGAAAA

**H132780266**

>spacer1

TTTGCCGATCCCCTTCCAGACCACCCTTTACA

>spacer2

TGCAGAGTTGTATCTTACCCTGTTTACGTTTC

>spacer3

CGTCACTTTCTGACATTTTATTCAGTTCGTTA

>spacer4

TCATTTCTGGACGGGGCTGTGTGACGAATACG

>spacer5

GGATATGTGAAGTTCAGGTAGCCCATTACGCA

>spacer6

TTGATCGAGAGTGCGAAGAGGCAGAACGGGCA

>spacer7

CAGGTTATGCGCAAAAATTAATTCATATTATA

>spacer8

GACGAGTTCTGGAAATGGTTAGCTGATAAAGA

>spacer9

CGTTCATCGGCAGCGTCACGCAATATGAAGAT

>spacer1

TGAGCAACGACAGTAAATAATTTTTCGTGCTG

>spacer2

CCAGTGGGCGTAGCCAGCTCATCGCTATTTTGC

>spacer3

CGTTGCGGATTATCGTTAAGACTGAAGGAAGT

>spacer4

TTGCAGGGCGATATTGTTGTTGGTGAATGGGA

>spacer5

CGTCGCGGAAAATTTCGCATTGACGATAAAGA

>spacer6

TTACGTGTTTATTCATCTGTTGCATTAGATTC

>spacer7

GAGGCGTACAGGCTGTTAGATGAGAAATTACC

>spacer8

ACGCCCCGAATGTGTTTGCCTCGCCCGCTGCC

>spacer9

TGGATTATCTGTATCTTACGGAAGTGGGCGCG

>spacer10

GTCGTTCATCAGGCACTACCGGCACTTTCTGG

>spacer11

ATATTCGCCGCTTTCCATTTACCGAACGTAAC

>spacer12

CCACGTTCGGCGATGTTGGCCCCATCGGTCCA

>spacer13

AAACGGTAGTGTTTTAAAACCGTTTCGAGGTGC

>spacer15

AGCCGTTTCCGCTAAATACCCCCGCAGTGATT

>spacer16

TTCTTGAATATGATTGCGGGTATATGTGGATA

>spacer17

TCTGGTTATAACATCGCAGCAAAATCAAAAGA

>spacer18

GCACTATTTCGAATGTCTCGACGCCAGATTTA

>spacer19

AACGAATTGAGACTATTAGAGATTATTCGCCT

>spacer20

GCAACCCATTAATTAACTAAGCAGTAATAAAC

>spacer21

TGACGAGGTGCGAGCGATGGTATCAAGGCCTA

>spacer22

GGTTAACCAGGGGTTTTTCCCCACTATTTCGC

>spacer23

AGGGGCGTTCCGCAGTCGACAAGGGCTGAAAA

**H132920685**

>spacer1

TTTTCAGCCCTTGTCGACTGCGGAACGCCCCT

>spacer2

GCGAAATAGTGGGGAAAAACCCCTGGTTAACC

>spacer3

TAGGCCTTGATACCATCGCTCGCACCTCGTCA

>spacer4

GTTTATTACTGCTTAGTTAATTAATGGGTTGC

>spacer5

AGGCGAATAATCTCTAATAGTCTCAATTCGTT

>spacer6

TAAATCTGGCGTCGAGACATTCGAAATAGTGC

>spacer7

TCTTTTGATTTTGCTGCGATGTTATAACCAGA

>spacer8

TATCCACATATACCCGCAATCATATTCAAGAA

>spacer9

AATCACTGCGGGGGTATTTAGCGGAAACGGCT

>spacer11

GCACCTCGAAACGGTTTTAAAACACTACCGTTT

>spacer12

TGGACCGATGGGGCCAACATCGCCGAACGTGG

>spacer13

GTTACGTTCGGTAAATGGAAAGCGGCGAATAT

>spacer14

CCAGAAAGTGCCGGTAGTGCCTGATGAACGAC

>spacer15

CGCGCCCACTTCCGTAAGATACAGATAATCCA

>spacer16

GGCAGCGGGCGAGGCAAACACATTCGGGGCGT

>spacer17

GGTAATTTCTCATCTAACAGCCTGTACGCCTC

>spacer18

GAATCTAATGCAACAGATGAATAAACACGTAA

>spacer19

TCTTTATCGTCAATGCGAAATTTTCCGCGACG

>spacer20

TCCCATTCACCAACAACAATATCGCCCTGCAA

>spacer21

ACTTCCTTCAGTCTTAACGATAATCCGCAACG

>spacer22

GCAAAATAGCGATGAGCTGGCTACGCCCACTGG

>spacer23

CAGCACGAAAAATTATTTACTGTCGTTGCTCA

>spacer1

ATCTTCATATTGCGTGACGCTGCCGATGAACG

>spacer2

TCTTTATCAGCTAACCATTTCCAGAACTCGTC

>spacer3

TATAATATGAATTAATTTTTGCGCATAACCTG

>spacer4

TGCCCGTTCTGCCTCTTCGCACTCTCGATCAA

>spacer5

TGCGTAATGGGCTACCTGAACTTCACATATCC

>spacer6

CGTATTCGTCACACAGCCCCGTCCAGAAATGA

>spacer7

TAACGAACTGAATAAAATGTCAGAAAGTGACG

>spacer8

GAAACGTAAACAGGGTAAGATACAACTCTGCA

>spacer9

TGTAAAGGGTGGTCTGGAAGGGGATCGGCAAA

H132940743

>spacer1

TTTGCCGATCCCCTTCCAGACCACCCTTTACA

>spacer2

TGCAGAGTTGTATCTTACCCTGTTTACGTTTC

>spacer3

CGTCACTTTCTGACATTTTATTCAGTTCGTTA

>spacer4

TCATTTCTGGACGGGGCTGTGTGACGAATACG

>spacer5

GGATATGTGAAGTTCAGGTAGCCCATTACGCA

>spacer6

TTGATCGAGAGTGCGAAGAGGCAGAACGGGCA

>spacer7

CAGGTTATGCGCAAAAATTAATTCATATTATA

>spacer8

GACGAGTTCTGGAAATGGTTAGCTGATAAAGA

>spacer9

CGTTCATCGGCAGCGTCACGCAATATGAAGAT

>spacer1

TGAGCAACGACAGTAAATAATTTTTCGTGCTG

>spacer2

CCAGTGGGCGTAGCCAGCTCATCGCTATTTTGC

>spacer3

CGTTGCGGATTATCGTTAAGACTGAAGGAAGT

>spacer4

TTGCAGGGCGATATTGTTGTTGGTGAATGGGA

>spacer5

CGTCGCGGAAAATTTCGCATTGACGATAAAGA

>spacer6

TTACGTGTTTATTCATCTGTTGCATTAGATTC

>spacer7

GAGGCGTACAGGCTGTTAGATGAGAAATTACC

>spacer8

ACGCCCCGAATGTGTTTGCCTCGCCCGCTGCC

>spacer9

TGGATTATCTGTATCTTACGGAAGTGGGCGCG

>spacer10

GTCGTTCATCAGGCACTACCGGCACTTTCTGG

>spacer11

ATATTCGCCGCTTTCCATTTACCGAACGTAAC

>spacer12

CCACGTTCGGCGATGTTGGCCCCATCGGTCCA

>spacer13

AAACGGTAGTGTTTTAAAACCGTTTCGAGGTGC

>spacer15

AGCCGTTTCCGCTAAATACCCCCGCAGTGATT

>spacer16

TTCTTGAATATGATTGCGGGTATATGTGGATA

>spacer17

TCTGGTTATAACATCGCAGCAAAATCAAAAGA

>spacer18

GCACTATTTCGAATGTCTCGACGCCAGATTTA

>spacer19

AACGAATTGAGACTATTAGAGATTATTCGCCT

>spacer20

GCAACCCATTAATTAACTAAGCAGTAATAAAC

>spacer21

TGACGAGGTGCGAGCGATGGTATCAAGGCCTA

>spacer22

GGTTAACCAGGGGTTTTTCCCCACTATTTCGC

>spacer23

AGGGGCGTTCCGCAGTCGACAAGGGCTGAAAA

**H132940744**

>spacer1

TTTGCCGATCCCCTTCCAGACCACCCTTTACA

>spacer2

TGCAGAGTTGTATCTTACCCTGTTTACGTTTC

>spacer3

CGTCACTTTCTGACATTTTATTCAGTTCGTTA

>spacer4

TCATTTCTGGACGGGGCTGTGTGACGAATACG

>spacer5

GGATATGTGAAGTTCAGGTAGCCCATTACGCA

>spacer6

TTGATCGAGAGTGCGAAGAGGCAGAACGGGCA

>spacer7

CAGGTTATGCGCAAAAATTAATTCATATTATA

>spacer8

GACGAGTTCTGGAAATGGTTAGCTGATAAAGA

>spacer9

CGTTCATCGGCAGCGTCACGCAATATGAAGAT

>spacer1

TGAGCAACGACAGTAAATAATTTTTCGTGCTG

>spacer2

CCAGTGGGCGTAGCCAGCTCATCGCTATTTTGC

>spacer3

CGTTGCGGATTATCGTTAAGACTGAAGGAAGT

>spacer4

TTGCAGGGCGATATTGTTGTTGGTGAATGGGA

>spacer5

CGTCGCGGAAAATTTCGCATTGACGATAAAGA

>spacer6

TTACGTGTTTATTCATCTGTTGCATTAGATTC

>spacer7

GAGGCGTACAGGCTGTTAGATGAGAAATTACC

>spacer8

ACGCCCCGAATGTGTTTGCCTCGCCCGCTGCC

>spacer9

TGGATTATCTGTATCTTACGGAAGTGGGCGCG

>spacer10

GTCGTTCATCAGGCACTACCGGCACTTTCTGG

>spacer11

ATATTCGCCGCTTTCCATTTACCGAACGTAAC

>spacer12

CCACGTTCGGCGATGTTGGCCCCATCGGTCCA

>spacer13

AAACGGTAGTGTTTTAAAACCGTTTCGAGGTGC

>spacer15

AGCCGTTTCCGCTAAATACCCCCGCAGTGATT

>spacer16

TTCTTGAATATGATTGCGGGTATATGTGGATA

>spacer17

TCTGGTTATAACATCGCAGCAAAATCAAAAGA

>spacer18

GCACTATTTCGAATGTCTCGACGCCAGATTTA

>spacer19

AACGAATTGAGACTATTAGAGATTATTCGCCT

>spacer20

GCAACCCATTAATTAACTAAGCAGTAATAAAC

>spacer21

TGACGAGGTGCGAGCGATGGTATCAAGGCCTA

>spacer22

GGTTAACCAGGGGTTTTTCCCCACTATTTCGC

>spacer23

AGGGGCGTTCCGCAGTCGACAAGGGCTGAAAA

**H132940745**

>spacer1

TTTTCAGCCCTTGTCGACTGCGGAACGCCCCT

>spacer2

GCGAAATAGTGGGGAAAAACCCCTGGTTAACC

>spacer3

TAGGCCTTGATACCATCGCTCGCACCTCGTCA

>spacer4

GTTTATTACTGCTTAGTTAATTAATGGGTTGC

>spacer5

AGGCGAATAATCTCTAATAGTCTCAATTCGTT

>spacer6

TAAATCTGGCGTCGAGACATTCGAAATAGTGC

>spacer7

TCTTTTGATTTTGCTGCGATGTTATAACCAGA

>spacer8

TATCCACATATACCCGCAATCATATTCAAGAA

>spacer9

AATCACTGCGGGGGTATTTAGCGGAAACGGCT

>spacer11

GCACCTCGAAACGGTTTTAAAACACTACCGTTT

>spacer12

TGGACCGATGGGGCCAACATCGCCGAACGTGG

>spacer13

GTTACGTTCGGTAAATGGAAAGCGGCGAATAT

>spacer14

CCAGAAAGTGCCGGTAGTGCCTGATGAACGAC

>spacer15

CGCGCCCACTTCCGTAAGATACAGATAATCCA

>spacer16

GGCAGCGGGCGAGGCAAACACATTCGGGGCGT

>spacer17

GGTAATTTCTCATCTAACAGCCTGTACGCCTC

>spacer18

GAATCTAATGCAACAGATGAATAAACACGTAA

>spacer19

TCTTTATCGTCAATGCGAAATTTTCCGCGACG

>spacer20

TCCCATTCACCAACAACAATATCGCCCTGCAA

>spacer21

ACTTCCTTCAGTCTTAACGATAATCCGCAACG

>spacer22

GCAAAATAGCGATGAGCTGGCTACGCCCACTGG

>spacer23

CAGCACGAAAAATTATTTACTGTCGTTGCTCA

>spacer1

ATCTTCATATTGCGTGACGCTGCCGATGAACG

>spacer2

TCTTTATCAGCTAACCATTTCCAGAACTCGTC

>spacer3

TATAATATGAATTAATTTTTGCGCATAACCTG

>spacer4

TGCCCGTTCTGCCTCTTCGCACTCTCGATCAA

>spacer5

TGCGTAATGGGCTACCTGAACTTCACATATCC

>spacer6

CGTATTCGTCACACAGCCCCGTCCAGAAATGA

>spacer7

TAACGAACTGAATAAAATGTCAGAAAGTGACG

>spacer8

GAAACGTAAACAGGGTAAGATACAACTCTGCA

>spacer9

TGTAAAGGGTGGTCTGGAAGGGGATCGGCAAA

**H132940746**

>spacer1

TTTTCAGCCCTTGTCGACTGCGGAACGCCCCT

>spacer2

GCGAAATAGTGGGGAAAAACCCCTGGTTAACC

>spacer3

TAGGCCTTGATACCATCGCTCGCACCTCGTCA

>spacer4

GTTTATTACTGCTTAGTTAATTAATGGGTTGC

>spacer5

AGGCGAATAATCTCTAATAGTCTCAATTCGTT

>spacer6

TAAATCTGGCGTCGAGACATTCGAAATAGTGC

>spacer7

TCTTTTGATTTTGCTGCGATGTTATAACCAGA

>spacer8

TATCCACATATACCCGCAATCATATTCAAGAA

>spacer9

AATCACTGCGGGGGTATTTAGCGGAAACGGCT

>spacer11

GCACCTCGAAACGGTTTTAAAACACTACCGTTT

>spacer12

TGGACCGATGGGGCCAACATCGCCGAACGTGG

>spacer13

GTTACGTTCGGTAAATGGAAAGCGGCGAATAT

>spacer14

CCAGAAAGTGCCGGTAGTGCCTGATGAACGAC

>spacer15

CGCGCCCACTTCCGTAAGATACAGATAATCCA

>spacer16

GGCAGCGGGCGAGGCAAACACATTCGGGGCGT

>spacer17

GGTAATTTCTCATCTAACAGCCTGTACGCCTC

>spacer18

GAATCTAATGCAACAGATGAATAAACACGTAA

>spacer19

TCTTTATCGTCAATGCGAAATTTTCCGCGACG

>spacer20

TCCCATTCACCAACAACAATATCGCCCTGCAA

>spacer21

ACTTCCTTCAGTCTTAACGATAATCCGCAACG

>spacer22

GCAAAATAGCGATGAGCTGGCTACGCCCACTGG

>spacer23

CAGCACGAAAAATTATTTACTGTCGTTGCTCA

>spacer1

ATCTTCATATTGCGTGACGCTGCCGATGAACG

>spacer2

TCTTTATCAGCTAACCATTTCCAGAACTCGTC

>spacer3

TATAATATGAATTAATTTTTGCGCATAACCTG

>spacer4

TGCCCGTTCTGCCTCTTCGCACTCTCGATCAA

>spacer5

TGCGTAATGGGCTACCTGAACTTCACATATCC

>spacer6

CGTATTCGTCACACAGCCCCGTCCAGAAATGA

>spacer7

TAACGAACTGAATAAAATGTCAGAAAGTGACG

>spacer8

GAAACGTAAACAGGGTAAGATACAACTCTGCA

>spacer9

TGTAAAGGGTGGTCTGGAAGGGGATCGGCAAA

**H132940748**

>spacer1

TTTGCCGATCCCCTTCCAGACCACCCTTTACA

>spacer2

TGCAGAGTTGTATCTTACCCTGTTTACGTTTC

>spacer3

CGTCACTTTCTGACATTTTATTCAGTTCGTTA

>spacer4

TCATTTCTGGACGGGGCTGTGTGACGAATACG

>spacer5

GGATATGTGAAGTTCAGGTAGCCCATTACGCA

>spacer6

TTGATCGAGAGTGCGAAGAGGCAGAACGGGCA

>spacer7

CAGGTTATGCGCAAAAATTAATTCATATTATA

>spacer8

GACGAGTTCTGGAAATGGTTAGCTGATAAAGA

>spacer9

CGTTCATCGGCAGCGTCACGCAATATGAAGAT

>spacer1

TGAGCAACGACAGTAAATAATTTTTCGTGCTG

>spacer2

CCAGTGGGCGTAGCCAGCTCATCGCTATTTTGC

>spacer3

CGTTGCGGATTATCGTTAAGACTGAAGGAAGT

>spacer4

TTGCAGGGCGATATTGTTGTTGGTGAATGGGA

>spacer5

CGTCGCGGAAAATTTCGCATTGACGATAAAGA

>spacer6

TTACGTGTTTATTCATCTGTTGCATTAGATTC

>spacer7

GAGGCGTACAGGCTGTTAGATGAGAAATTACC

>spacer8

ACGCCCCGAATGTGTTTGCCTCGCCCGCTGCC

>spacer9

TGGATTATCTGTATCTTACGGAAGTGGGCGCG

>spacer10

GTCGTTCATCAGGCACTACCGGCACTTTCTGG

>spacer11

ATATTCGCCGCTTTCCATTTACCGAACGTAAC

>spacer12

CCACGTTCGGCGATGTTGGCCCCATCGGTCCA

>spacer13

AAACGGTAGTGTTTTAAAACCGTTTCGAGGTGC

>spacer15

AGCCGTTTCCGCTAAATACCCCCGCAGTGATT

>spacer16

TTCTTGAATATGATTGCGGGTATATGTGGATA

>spacer17

TCTGGTTATAACATCGCAGCAAAATCAAAAGA

>spacer18

GCACTATTTCGAATGTCTCGACGCCAGATTTA

>spacer19

AACGAATTGAGACTATTAGAGATTATTCGCCT

>spacer20

GCAACCCATTAATTAACTAAGCAGTAATAAAC

>spacer21

TGACGAGGTGCGAGCGATGGTATCAAGGCCTA

>spacer22

GGTTAACCAGGGGTTTTTCCCCACTATTTCGC

>spacer23

AGGGGCGTTCCGCAGTCGACAAGGGCTGAAAA

**H132940749**

>spacer1

TTTGCCGATCCCCTTCCAGACCACCCTTTACA

>spacer2

TGCAGAGTTGTATCTTACCCTGTTTACGTTTC

>spacer3

CGTCACTTTCTGACATTTTATTCAGTTCGTTA

>spacer4

TCATTTCTGGACGGGGCTGTGTGACGAATACG

>spacer5

GGATATGTGAAGTTCAGGTAGCCCATTACGCA

>spacer6

TTGATCGAGAGTGCGAAGAGGCAGAACGGGCA

>spacer7

CAGGTTATGCGCAAAAATTAATTCATATTATA

>spacer8

GACGAGTTCTGGAAATGGTTAGCTGATAAAGA

>spacer9

CGTTCATCGGCAGCGTCACGCAATATGAAGAT

>spacer1

TGAGCAACGACAGTAAATAATTTTTCGTGCTG

>spacer2

CCAGTGGGCGTAGCCAGCTCATCGCTATTTTGC

>spacer3

CGTTGCGGATTATCGTTAAGACTGAAGGAAGT

>spacer4

TTGCAGGGCGATATTGTTGTTGGTGAATGGGA

>spacer5

CGTCGCGGAAAATTTCGCATTGACGATAAAGA

>spacer6

TTACGTGTTTATTCATCTGTTGCATTAGATTC

>spacer7

GAGGCGTACAGGCTGTTAGATGAGAAATTACC

>spacer8

ACGCCCCGAATGTGTTTGCCTCGCCCGCTGCC

>spacer9

TGGATTATCTGTATCTTACGGAAGTGGGCGCG

>spacer10

GTCGTTCATCAGGCACTACCGGCACTTTCTGG

>spacer11

ATATTCGCCGCTTTCCATTTACCGAACGTAAC

>spacer12

CCACGTTCGGCGATGTTGGCCCCATCGGTCCA

>spacer13

AAACGGTAGTGTTTTAAAACCGTTTCGAGGTGC

>spacer15

AGCCGTTTCCGCTAAATACCCCCGCAGTGATT

>spacer16

TTCTTGAATATGATTGCGGGTATATGTGGATA

>spacer17

TCTGGTTATAACATCGCAGCAAAATCAAAAGA

>spacer18

GCACTATTTCGAATGTCTCGACGCCAGATTTA

>spacer19

AACGAATTGAGACTATTAGAGATTATTCGCCT

>spacer20

GCAACCCATTAATTAACTAAGCAGTAATAAAC

>spacer21

TGACGAGGTGCGAGCGATGGTATCAAGGCCTA

>spacer22

GGTTAACCAGGGGTTTTTCCCCACTATTTCGC

>spacer23

AGGGGCGTTCCGCAGTCGACAAGGGCTGAAAA

**H132940750**

>spacer1

TTTTCAGCCCTTGTCGACTGCGGAACGCCCCT

>spacer2

GCGAAATAGTGGGGAAAAACCCCTGGTTAACC

>spacer3

TAGGCCTTGATACCATCGCTCGCACCTCGTCA

>spacer4

GTTTATTACTGCTTAGTTAATTAATGGGTTGC

>spacer5

AGGCGAATAATCTCTAATAGTCTCAATTCGTT

>spacer6

TAAATCTGGCGTCGAGACATTCGAAATAGTGC

>spacer7

TCTTTTGATTTTGCTGCGATGTTATAACCAGA

>spacer8

TATCCACATATACCCGCAATCATATTCAAGAA

>spacer9

AATCACTGCGGGGGTATTTAGCGGAAACGGCT

>spacer11

GCACCTCGAAACGGTTTTAAAACACTACCGTTT

>spacer12

TGGACCGATGGGGCCAACATCGCCGAACGTGG

>spacer13

GTTACGTTCGGTAAATGGAAAGCGGCGAATAT

>spacer14

CCAGAAAGTGCCGGTAGTGCCTGATGAACGAC

>spacer15

CGCGCCCACTTCCGTAAGATACAGATAATCCA

>spacer16

GGCAGCGGGCGAGGCAAACACATTCGGGGCGT

>spacer17

GGTAATTTCTCATCTAACAGCCTGTACGCCTC

>spacer18

GAATCTAATGCAACAGATGAATAAACACGTAA

>spacer19

TCTTTATCGTCAATGCGAAATTTTCCGCGACG

>spacer20

TCCCATTCACCAACAACAATATCGCCCTGCAA

>spacer21

ACTTCCTTCAGTCTTAACGATAATCCGCAACG

>spacer22

GCAAAATAGCGATGAGCTGGCTACGCCCACTGG

>spacer23

CAGCACGAAAAATTATTTACTGTCGTTGCTCA

>spacer1

ATCTTCATATTGCGTGACGCTGCCGATGAACG

>spacer2

TCTTTATCAGCTAACCATTTCCAGAACTCGTC

>spacer3

TATAATATGAATTAATTTTTGCGCATAACCTG

>spacer4

TGCCCGTTCTGCCTCTTCGCACTCTCGATCAA

>spacer5

TGCGTAATGGGCTACCTGAACTTCACATATCC

>spacer6

CGTATTCGTCACACAGCCCCGTCCAGAAATGA

>spacer7

TAACGAACTGAATAAAATGTCAGAAAGTGACG

>spacer8

GAAACGTAAACAGGGTAAGATACAACTCTGCA

>spacer9

TGTAAAGGGTGGTCTGGAAGGGGATCGGCAAA

**H132940751**

>spacer1

TTTGCCGATCCCCTTCCAGACCACCCTTTACA

>spacer2

TGCAGAGTTGTATCTTACCCTGTTTACGTTTC

>spacer3

CGTCACTTTCTGACATTTTATTCAGTTCGTTA

>spacer4

TCATTTCTGGACGGGGCTGTGTGACGAATACG

>spacer5

GGATATGTGAAGTTCAGGTAGCCCATTACGCA

>spacer6

TTGATCGAGAGTGCGAAGAGGCAGAACGGGCA

>spacer7

CAGGTTATGCGCAAAAATTAATTCATATTATA

>spacer8

GACGAGTTCTGGAAATGGTTAGCTGATAAAGA

>spacer9

CGTTCATCGGCAGCGTCACGCAATATGAAGAT

>spacer1

TGAGCAACGACAGTAAATAATTTTTCGTGCTG

>spacer2

CCAGTGGGCGTAGCCAGCTCATCGCTATTTTGC

>spacer3

CGTTGCGGATTATCGTTAAGACTGAAGGAAGT

>spacer4

TTGCAGGGCGATATTGTTGTTGGTGAATGGGA

>spacer5

CGTCGCGGAAAATTTCGCATTGACGATAAAGA

>spacer6

TTACGTGTTTATTCATCTGTTGCATTAGATTC

>spacer7

GAGGCGTACAGGCTGTTAGATGAGAAATTACC

>spacer8

ACGCCCCGAATGTGTTTGCCTCGCCCGCTGCC

>spacer9

TGGATTATCTGTATCTTACGGAAGTGGGCGCG

>spacer10

GTCGTTCATCAGGCACTACCGGCACTTTCTGG

>spacer11

ATATTCGCCGCTTTCCATTTACCGAACGTAAC

>spacer12

CCACGTTCGGCGATGTTGGCCCCATCGGTCCA

>spacer13

AAACGGTAGTGTTTTAAAACCGTTTCGAGGTGC

>spacer15

AGCCGTTTCCGCTAAATACCCCCGCAGTGATT

>spacer16

TTCTTGAATATGATTGCGGGTATATGTGGATA

>spacer17

TCTGGTTATAACATCGCAGCAAAATCAAAAGA

>spacer18

GCACTATTTCGAATGTCTCGACGCCAGATTTA

>spacer19

AACGAATTGAGACTATTAGAGATTATTCGCCT

>spacer20

GCAACCCATTAATTAACTAAGCAGTAATAAAC

>spacer21

TGACGAGGTGCGAGCGATGGTATCAAGGCCTA

>spacer22

GGTTAACCAGGGGTTTTTCCCCACTATTTCGC

>spacer23

AGGGGCGTTCCGCAGTCGACAAGGGCTGAAAA

**H132940753**

>spacer1

TGAGCAACGACAGTAAATAATTTTTCGTGCTG

>spacer2

CCAGTGGGCGTAGCCAGCTCATCGCTATTTTGC

>spacer3

CGTTGCGGATTATCGTTAAGACTGAAGGAAGT

>spacer4

TTGCAGGGCGATATTGTTGTTGGTGAATGGGA

>spacer5

CGTCGCGGAAAATTTCGCATTGACGATAAAGA

>spacer6

TTACGTGTTTATTCATCTGTTGCATTAGATTC

>spacer7

GAGGCGTACAGGCTGTTAGATGAGAAATTACC

>spacer8

ACGCCCCGAATGTGTTTGCCTCGCCCGCTGCC

>spacer9

TGGATTATCTGTATCTTACGGAAGTGGGCGCG

>spacer10

GTCGTTCATCAGGCACTACCGGCACTTTCTGG

>spacer11

ATATTCGCCGCTTTCCATTTACCGAACGTAAC

>spacer12

CCACGTTCGGCGATGTTGGCCCCATCGGTCCA

>spacer13

AAACGGTAGTGTTTTAAAACCGTTTCGAGGTGC

>spacer15

AGCCGTTTCCGCTAAATACCCCCGCAGTGATT

>spacer16

TTCTTGAATATGATTGCGGGTATATGTGGATA

>spacer17

TCTGGTTATAACATCGCAGCAAAATCAAAAGA

>spacer18

GCACTATTTCGAATGTCTCGACGCCAGATTTA

>spacer19

AACGAATTGAGACTATTAGAGATTATTCGCCT

>spacer20

GCAACCCATTAATTAACTAAGCAGTAATAAAC

>spacer21

TGACGAGGTGCGAGCGATGGTATCAAGGCCTA

>spacer22

GGTTAACCAGGGGTTTTTCCCCACTATTTCGC

>spacer23

AGGGGCGTTCCGCAGTCGACAAGGGCTGAAAA

>spacer1

ACCGTTTGCCGATCCCCTTCCAGACCACCCTTTACA

>spacer2

ACCGTGCAGAGTTGTATCTTACCCTGTTTACGTTTC

>spacer3

GCCGCGTCACTTTCTGACATTTTATTCAGTTCGTTA

>spacer4

ACCGTCATTTCTGGACGGGGCTGTGTGACGAATACG

>spacer5

ACCGGGATATGTGAAGTTCAGGTAGCCCATTACGCA

>spacer6

ACCGTTGATCGAGAGTGCGAAGAGGCAGAACGGGCA

>spacer7

ACCGCAGGTTATGCGCAAAAATTAATTCATATTATA

>spacer8

ACCGGACGAGTTCTGGAAATGGTTAGCTGATAAAGA

>spacer9

ACCGCGTTCATCGGCAGCGTCACGCAATATGAAGAT

**H132940754**

>spacer1

ATCTTCATATTGCGTGACGCTGCCGATGAACG

>spacer2

TCTTTATCAGCTAACCATTTCCAGAACTCGTC

>spacer3

TATAATATGAATTAATTTTTGCGCATAACCTG

>spacer4

TGCCCGTTCTGCCTCTTCGCACTCTCGATCAA

>spacer5

TGCGTAATGGGCTACCTGAACTTCACATATCC

>spacer6

CGTATTCGTCACACAGCCCCGTCCAGAAATGA

>spacer7

TAACGAACTGAATAAAATGTCAGAAAGTGACG

>spacer8

GAAACGTAAACAGGGTAAGATACAACTCTGCA

>spacer9

TGTAAAGGGTGGTCTGGAAGGGGATCGGCAAA

>spacer1

TGAGCAACGACAGTAAATAATTTTTCGTGCTG

>spacer2

CCAGTGGGCGTAGCCAGCTCATCGCTATTTTGC

>spacer3

CGTTGCGGATTATCGTTAAGACTGAAGGAAGT

>spacer4

TTGCAGGGCGATATTGTTGTTGGTGAATGGGA

>spacer5

CGTCGCGGAAAATTTCGCATTGACGATAAAGA

>spacer6

TTACGTGTTTATTCATCTGTTGCATTAGATTC

>spacer7

GAGGCGTACAGGCTGTTAGATGAGAAATTACC

>spacer8

ACGCCCCGAATGTGTTTGCCTCGCCCGCTGCC

>spacer9

TGGATTATCTGTATCTTACGGAAGTGGGCGCG

>spacer10

GTCGTTCATCAGGCACTACCGGCACTTTCTGG

>spacer11

ATATTCGCCGCTTTCCATTTACCGAACGTAAC

>spacer12

CCACGTTCGGCGATGTTGGCCCCATCGGTCCA

>spacer13

AAACGGTAGTGTTTTAAAACCGTTTCGAGGTGC

>spacer15

AGCCGTTTCCGCTAAATACCCCCGCAGTGATT

>spacer16

TTCTTGAATATGATTGCGGGTATATGTGGATA

>spacer17

TCTGGTTATAACATCGCAGCAAAATCAAAAGA

>spacer18

GCACTATTTCGAATGTCTCGACGCCAGATTTA

>spacer19

AACGAATTGAGACTATTAGAGATTATTCGCCT

>spacer20

GCAACCCATTAATTAACTAAGCAGTAATAAAC

>spacer21

TGACGAGGTGCGAGCGATGGTATCAAGGCCTA

>spacer22

GGTTAACCAGGGGTTTTTCCCCACTATTTCGC

>spacer23

AGGGGCGTTCCGCAGTCGACAAGGGCTGAAAA

**H132940756**

>spacer1

TTTTCAGCCCTTGTCGACTGCGGAACGCCCCT

>spacer2

GCGAAATAGTGGGGAAAAACCCCTGGTTAACC

>spacer3

TAGGCCTTGATACCATCGCTCGCACCTCGTCA

>spacer4

GTTTATTACTGCTTAGTTAATTAATGGGTTGC

>spacer5

AGGCGAATAATCTCTAATAGTCTCAATTCGTT

>spacer6

TAAATCTGGCGTCGAGACATTCGAAATAGTGC

>spacer7

TCTTTTGATTTTGCTGCGATGTTATAACCAGA

>spacer8

TATCCACATATACCCGCAATCATATTCAAGAA

>spacer9

AATCACTGCGGGGGTATTTAGCGGAAACGGCT

>spacer11

GCACCTCGAAACGGTTTTAAAACACTACCGTTT

>spacer12

TGGACCGATGGGGCCAACATCGCCGAACGTGG

>spacer13

GTTACGTTCGGTAAATGGAAAGCGGCGAATAT

>spacer14

CCAGAAAGTGCCGGTAGTGCCTGATGAACGAC

>spacer15

CGCGCCCACTTCCGTAAGATACAGATAATCCA

>spacer16

GGCAGCGGGCGAGGCAAACACATTCGGGGCGT

>spacer17

GGTAATTTCTCATCTAACAGCCTGTACGCCTC

>spacer18

GAATCTAATGCAACAGATGAATAAACACGTAA

>spacer19

TCTTTATCGTCAATGCGAAATTTTCCGCGACG

>spacer20

TCCCATTCACCAACAACAATATCGCCCTGCAA

>spacer21

ACTTCCTTCAGTCTTAACGATAATCCGCAACG

>spacer22

GCAAAATAGCGATGAGCTGGCTACGCCCACTGG

>spacer23

CAGCACGAAAAATTATTTACTGTCGTTGCTCA

>spacer1

ATCTTCATATTGCGTGACGCTGCCGATGAACG

>spacer2

TCTTTATCAGCTAACCATTTCCAGAACTCGTC

>spacer3

TATAATATGAATTAATTTTTGCGCATAACCTG

>spacer4

TGCCCGTTCTGCCTCTTCGCACTCTCGATCAA

>spacer5

TGCGTAATGGGCTACCTGAACTTCACATATCC

>spacer6

CGTATTCGTCACACAGCCCCGTCCAGAAATGA

>spacer7

TAACGAACTGAATAAAATGTCAGAAAGTGACG

>spacer8

GAAACGTAAACAGGGTAAGATACAACTCTGCA

>spacer9

TGTAAAGGGTGGTCTGGAAGGGGATCGGCAAA

**H132960590**

>spacer1

TTTGCCGATCCCCTTCCAGACCACCCTTTACA

>spacer2

TGCAGAGTTGTATCTTACCCTGTTTACGTTTC

>spacer3

CGTCACTTTCTGACATTTTATTCAGTTCGTTA

>spacer4

TCATTTCTGGACGGGGCTGTGTGACGAATACG

>spacer5

GGATATGTGAAGTTCAGGTAGCCCATTACGCA

>spacer6

TTGATCGAGAGTGCGAAGAGGCAGAACGGGCA

>spacer7

CAGGTTATGCGCAAAAATTAATTCATATTATA

>spacer8

GACGAGTTCTGGAAATGGTTAGCTGATAAAGA

>spacer9

CGTTCATCGGCAGCGTCACGCAATATGAAGAT

>spacer1

TGAGCAACGACAGTAAATAATTTTTCGTGCTG

>spacer2

CCAGTGGGCGTAGCCAGCTCATCGCTATTTTGC

>spacer3

CGTTGCGGATTATCGTTAAGACTGAAGGAAGT

>spacer4

TTGCAGGGCGATATTGTTGTTGGTGAATGGGA

>spacer5

CGTCGCGGAAAATTTCGCATTGACGATAAAGA

>spacer6

TTACGTGTTTATTCATCTGTTGCATTAGATTC

>spacer7

GAGGCGTACAGGCTGTTAGATGAGAAATTACC

>spacer8

ACGCCCCGAATGTGTTTGCCTCGCCCGCTGCC

>spacer9

TGGATTATCTGTATCTTACGGAAGTGGGCGCG

>spacer10

GTCGTTCATCAGGCACTACCGGCACTTTCTGG

>spacer11

ATATTCGCCGCTTTCCATTTACCGAACGTAAC

>spacer12

CCACGTTCGGCGATGTTGGCCCCATCGGTCCA

>spacer13

AAACGGTAGTGTTTTAAAACCGTTTCGAGGTGC

>spacer15

AGCCGTTTCCGCTAAATACCCCCGCAGTGATT

>spacer16

TTCTTGAATATGATTGCGGGTATATGTGGATA

>spacer17

TCTGGTTATAACATCGCAGCAAAATCAAAAGA

>spacer18

GCACTATTTCGAATGTCTCGACGCCAGATTTA

>spacer19

AACGAATTGAGACTATTAGAGATTATTCGCCT

>spacer20

GCAACCCATTAATTAACTAAGCAGTAATAAAC

>spacer21

TGACGAGGTGCGAGCGATGGTATCAAGGCCTA

>spacer22

GGTTAACCAGGGGTTTTTCCCCACTATTTCGC

>spacer23

AGGGGCGTTCCGCAGTCGACAAGGGCTGAAAA

**H132980531**

>spacer1

TTTTCAGCCCTTGTCGACTGCGGAACGCCCCT

>spacer2

GCGAAATAGTGGGGAAAAACCCCTGGTTAACC

>spacer3

TAGGCCTTGATACCATCGCTCGCACCTCGTCA

>spacer4

GTTTATTACTGCTTAGTTAATTAATGGGTTGC

>spacer5

AGGCGAATAATCTCTAATAGTCTCAATTCGTT

>spacer6

TAAATCTGGCGTCGAGACATTCGAAATAGTGC

>spacer7

TCTTTTGATTTTGCTGCGATGTTATAACCAGA

>spacer8

TATCCACATATACCCGCAATCATATTCAAGAA

>spacer9

AATCACTGCGGGGGTATTTAGCGGAAACGGCT

>spacer11

GCACCTCGAAACGGTTTTAAAACACTACCGTTT

>spacer12

TGGACCGATGGGGCCAACATCGCCGAACGTGG

>spacer13

GTTACGTTCGGTAAATGGAAAGCGGCGAATAT

>spacer14

CCAGAAAGTGCCGGTAGTGCCTGATGAACGAC

>spacer15

CGCGCCCACTTCCGTAAGATACAGATAATCCA

>spacer16

GGCAGCGGGCGAGGCAAACACATTCGGGGCGT

>spacer17

GGTAATTTCTCATCTAACAGCCTGTACGCCTC

>spacer18

GAATCTAATGCAACAGATGAATAAACACGTAA

>spacer19

TCTTTATCGTCAATGCGAAATTTTCCGCGACG

>spacer20

TCCCATTCACCAACAACAATATCGCCCTGCAA

>spacer21

ACTTCCTTCAGTCTTAACGATAATCCGCAACG

>spacer22

GCAAAATAGCGATGAGCTGGCTACGCCCACTGG

>spacer23

CAGCACGAAAAATTATTTACTGTCGTTGCTCA

>spacer1

ATCTTCATATTGCGTGACGCTGCCGATGAACG

>spacer2

TCTTTATCAGCTAACCATTTCCAGAACTCGTC

>spacer3

TATAATATGAATTAATTTTTGCGCATAACCTG

>spacer4

TGCCCGTTCTGCCTCTTCGCACTCTCGATCAA

>spacer5

TGCGTAATGGGCTACCTGAACTTCACATATCC

>spacer6

CGTATTCGTCACACAGCCCCGTCCAGAAATGA

>spacer7

TAACGAACTGAATAAAATGTCAGAAAGTGACG

>spacer8

GAAACGTAAACAGGGTAAGATACAACTCTGCA

>spacer9

TGTAAAGGGTGGTCTGGAAGGGGATCGGCAAA

**H133000645**

>spacer1

TTTTCAGCCCTTGTCGACTGCGGAACGCCCCT

>spacer2

GCGAAATAGTGGGGAAAAACCCCTGGTTAACC

>spacer3

TAGGCCTTGATACCATCGCTCGCACCTCGTCA

>spacer4

GTTTATTACTGCTTAGTTAATTAATGGGTTGC

>spacer5

AGGCGAATAATCTCTAATAGTCTCAATTCGTT

>spacer6

TAAATCTGGCGTCGAGACATTCGAAATAGTGC

>spacer7

TCTTTTGATTTTGCTGCGATGTTATAACCAGA

>spacer8

TATCCACATATACCCGCAATCATATTCAAGAA

>spacer9

AATCACTGCGGGGGTATTTAGCGGAAACGGCT

>spacer11

GCACCTCGAAACGGTTTTAAAACACTACCGTTT

>spacer12

TGGACCGATGGGGCCAACATCGCCGAACGTGG

>spacer13

GTTACGTTCGGTAAATGGAAAGCGGCGAATAT

>spacer14

CCAGAAAGTGCCGGTAGTGCCTGATGAACGAC

>spacer15

CGCGCCCACTTCCGTAAGATACAGATAATCCA

>spacer16

GGCAGCGGGCGAGGCAAACACATTCGGGGCGT

>spacer17

GGTAATTTCTCATCTAACAGCCTGTACGCCTC

>spacer18

GAATCTAATGCAACAGATGAATAAACACGTAA

>spacer19

TCTTTATCGTCAATGCGAAATTTTCCGCGACG

>spacer20

TCCCATTCACCAACAACAATATCGCCCTGCAA

>spacer21

ACTTCCTTCAGTCTTAACGATAATCCGCAACG

>spacer22

GCAAAATAGCGATGAGCTGGCTACGCCCACTGG

>spacer23

CAGCACGAAAAATTATTTACTGTCGTTGCTCA

>spacer1

ATCTTCATATTGCGTGACGCTGCCGATGAACG

>spacer2

TCTTTATCAGCTAACCATTTCCAGAACTCGTC

>spacer3

TATAATATGAATTAATTTTTGCGCATAACCTG

>spacer4

TGCCCGTTCTGCCTCTTCGCACTCTCGATCAA

>spacer5

TGCGTAATGGGCTACCTGAACTTCACATATCC

>spacer6

CGTATTCGTCACACAGCCCCGTCCAGAAATGA

>spacer7

TAACGAACTGAATAAAATGTCAGAAAGTGACG

>spacer8

GAAACGTAAACAGGGTAAGATACAACTCTGCA

>spacer9

TGTAAAGGGTGGTCTGGAAGGGGATCGGCAAA

**H133000654**

>spacer1

TTTGCCGATCCCCTTCCAGACCACCCTTTACA

>spacer2

TGCAGAGTTGTATCTTACCCTGTTTACGTTTC

>spacer3

CGTCACTTTCTGACATTTTATTCAGTTCGTTA

>spacer4

TCATTTCTGGACGGGGCTGTGTGACGAATACG

>spacer5

GGATATGTGAAGTTCAGGTAGCCCATTACGCA

>spacer6

TTGATCGAGAGTGCGAAGAGGCAGAACGGGCA

>spacer7

CAGGTTATGCGCAAAAATTAATTCATATTATA

>spacer8

GACGAGTTCTGGAAATGGTTAGCTGATAAAGA

>spacer9

CGTTCATCGGCAGCGTCACGCAATATGAAGAT

>spacer1

TGAGCAACGACAGTAAATAATTTTTCGTGCTG

>spacer2

CCAGTGGGCGTAGCCAGCTCATCGCTATTTTGC

>spacer3

CGTTGCGGATTATCGTTAAGACTGAAGGAAGT

>spacer4

TTGCAGGGCGATATTGTTGTTGGTGAATGGGA

>spacer5

CGTCGCGGAAAATTTCGCATTGACGATAAAGA

>spacer6

TTACGTGTTTATTCATCTGTTGCATTAGATTC

>spacer7

GAGGCGTACAGGCTGTTAGATGAGAAATTACC

>spacer8

ACGCCCCGAATGTGTTTGCCTCGCCCGCTGCC

>spacer9

TGGATTATCTGTATCTTACGGAAGTGGGCGCG

>spacer10

GTCGTTCATCAGGCACTACCGGCACTTTCTGG

>spacer11

ATATTCGCCGCTTTCCATTTACCGAACGTAAC

>spacer12

CCACGTTCGGCGATGTTGGCCCCATCGGTCCA

>spacer13

AAACGGTAGTGTTTTAAAACCGTTTCGAGGTGC

>spacer15

AGCCGTTTCCGCTAAATACCCCCGCAGTGATT

>spacer16

TTCTTGAATATGATTGCGGGTATATGTGGATA

>spacer17

TCTGGTTATAACATCGCAGCAAAATCAAAAGA

>spacer18

GCACTATTTCGAATGTCTCGACGCCAGATTTA

>spacer19

AACGAATTGAGACTATTAGAGATTATTCGCCT

>spacer20

GCAACCCATTAATTAACTAAGCAGTAATAAAC

>spacer21

TGACGAGGTGCGAGCGATGGTATCAAGGCCTA

>spacer22

GGTTAACCAGGGGTTTTTCCCCACTATTTCGC

>spacer23

AGGGGCGTTCCGCAGTCGACAAGGGCTGAAAA

**H133040470**

>spacer1

TTTTCAGCCCTTGTCGACTGCGGAACGCCCCT

>spacer2

GCGAAATAGTGGGGAAAAACCCCTGGTTAACC

>spacer3

TAGGCCTTGATACCATCGCTCGCACCTCGTCA

>spacer4

GTTTATTACTGCTTAGTTAATTAATGGGTTGC

>spacer5

AGGCGAATAATCTCTAATAGTCTCAATTCGTT

>spacer6

TAAATCTGGCGTCGAGACATTCGAAATAGTGC

>spacer7

TCTTTTGATTTTGCTGCGATGTTATAACCAGA

>spacer8

TATCCACATATACCCGCAATCATATTCAAGAA

>spacer9

AATCACTGCGGGGGTATTTAGCGGAAACGGCT

>spacer11

GCACCTCGAAACGGTTTTAAAACACTACCGTTT

>spacer12

TGGACCGATGGGGCCAACATCGCCGAACGTGG

>spacer13

GTTACGTTCGGTAAATGGAAAGCGGCGAATAT

>spacer14

CCAGAAAGTGCCGGTAGTGCCTGATGAACGAC

>spacer15

CGCGCCCACTTCCGTAAGATACAGATAATCCA

>spacer16

GGCAGCGGGCGAGGCAAACACATTCGGGGCGT

>spacer17

GGTAATTTCTCATCTAACAGCCTGTACGCCTC

>spacer18

GAATCTAATGCAACAGATGAATAAACACGTAA

>spacer19

TCTTTATCGTCAATGCGAAATTTTCCGCGACG

>spacer20

TCCCATTCACCAACAACAATATCGCCCTGCAA

>spacer21

ACTTCCTTCAGTCTTAACGATAATCCGCAACG

>spacer22

GCAAAATAGCGATGAGCTGGCTACGCCCACTGG

>spacer23

CAGCACGAAAAATTATTTACTGTCGTTGCTCA

>spacer1

ATCTTCATATTGCGTGACGCTGCCGATGAACG

>spacer2

TCTTTATCAGCTAACCATTTCCAGAACTCGTC

>spacer3

TATAATATGAATTAATTTTTGCGCATAACCTG

>spacer4

TGCCCGTTCTGCCTCTTCGCACTCTCGATCAA

>spacer5

TGCGTAATGGGCTACCTGAACTTCACATATCC

>spacer6

CGTATTCGTCACACAGCCCCGTCCAGAAATGA

>spacer7

TAACGAACTGAATAAAATGTCAGAAAGTGACG

>spacer8

GAAACGTAAACAGGGTAAGATACAACTCTGCA

>spacer9

TGTAAAGGGTGGTCTGGAAGGGGATCGGCAAA

**H122060375**

>spacer1

TTTGCCGATCCCCTTCCAGACCACCCTTTACA

>spacer2

TGCAGAGTTGTATCTTACCCTGTTTACGTTTC

>spacer3

CGTCACTTTCTGACATTTTATTCAGTTCGTTA

>spacer4

TCATTTCTGGACGGGGCTGTGTGACGAATACG

>spacer5

GGATATGTGAAGTTCAGGTAGCCCATTACGCA

>spacer6

TTGATCGAGAGTGCGAAGAGGCAGAACGGGCA

>spacer7

CAGGTTATGCGCAAAAATTAATTCATATTATA

>spacer8

GACGAGTTCTGGAAATGGTTAGCTGATAAAGA

>spacer9

CGTTCATCGGCAGCGTCACGCAATATGAAGAT

>spacer1

TGAGCAACGACAGTAAATAATTTTTCGTGCTG

>spacer2

CCAGTGGGCGTAGCCAGCTCATCGCTATTTTGC

>spacer3

CGTTGCGGATTATCGTTAAGACTGAAGGAAGT

>spacer4

TTGCAGGGCGATATTGTTGTTGGTGAATGGGA

>spacer5

CGTCGCGGAAAATTTCGCATTGACGATAAAGA

>spacer6

TTACGTGTTTATTCATCTGTTGCATTAGATTC

>spacer7

GAGGCGTACAGGCTGTTAGATGAGAAATTACC

>spacer8

ACGCCCCGAATGTGTTTGCCTCGCCCGCTGCC

>spacer9

TGGATTATCTGTATCTTACGGAAGTGGGCGCG

>spacer10

GTCGTTCATCAGGCACTACCGGCACTTTCTGG

>spacer11

ATATTCGCCGCTTTCCATTTACCGAACGTAAC

>spacer12

CCACGTTCGGCGATGTTGGCCCCATCGGTCCA

>spacer13

AAACGGTAGTGTTTTAAAACCGTTTCGAGGTGC

>spacer15

AGCCGTTTCCGCTAAATACCCCCGCAGTGATT

>spacer16

TTCTTGAATATGATTGCGGGTATATGTGGATA

>spacer17

TCTGGTTATAACATCGCAGCAAAATCAAAAGA

>spacer18

GCACTATTTCGAATGTCTCGACGCCAGATTTA

>spacer19

AACGAATTGAGACTATTAGAGATTATTCGCCT

>spacer20

GCAACCCATTAATTAACTAAGCAGTAATAAAC

>spacer21

TGACGAGGTGCGAGCGATGGTATCAAGGCCTA

>spacer22

GGTTAACCAGGGGTTTTTCCCCACTATTTCGC

>spacer23

AGGGGCGTTCCGCAGTCGACAAGGGCTGAAAA

**H133060376**

>spacer1

TTTTCAGCCCTTGTCGACTGCGGAACGCCCCT

>spacer2

GCGAAATAGTGGGGAAAAACCCCTGGTTAACC

>spacer3

TAGGCCTTGATACCATCGCTCGCACCTCGTCA

>spacer4

GTTTATTACTGCTTAGTTAATTAATGGGTTGC

>spacer5

AGGCGAATAATCTCTAATAGTCTCAATTCGTT

>spacer6

TAAATCTGGCGTCGAGACATTCGAAATAGTGC

>spacer7

TCTTTTGATTTTGCTGCGATGTTATAACCAGA

>spacer8

TATCCACATATACCCGCAATCATATTCAAGAA

>spacer9

AATCACTGCGGGGGTATTTAGCGGAAACGGCT

>spacer11

GCACCTCGAAACGGTTTTAAAACACTACCGTTT

>spacer12

TGGACCGATGGGGCCAACATCGCCGAACGTGG

>spacer13

GTTACGTTCGGTAAATGGAAAGCGGCGAATAT

>spacer14

CCAGAAAGTGCCGGTAGTGCCTGATGAACGAC

>spacer15

CGCGCCCACTTCCGTAAGATACAGATAATCCA

>spacer16

GGCAGCGGGCGAGGCAAACACATTCGGGGCGT

>spacer17

GGTAATTTCTCATCTAACAGCCTGTACGCCTC

>spacer18

GAATCTAATGCAACAGATGAATAAACACGTAA

>spacer19

TCTTTATCGTCAATGCGAAATTTTCCGCGACG

>spacer20

TCCCATTCACCAACAACAATATCGCCCTGCAA

>spacer21

ACTTCCTTCAGTCTTAACGATAATCCGCAACG

>spacer22

GCAAAATAGCGATGAGCTGGCTACGCCCACTGG

>spacer23

CAGCACGAAAAATTATTTACTGTCGTTGCTCA

>spacer1

ATCTTCATATTGCGTGACGCTGCCGATGAACG

>spacer2

TCTTTATCAGCTAACCATTTCCAGAACTCGTC

>spacer3

TATAATATGAATTAATTTTTGCGCATAACCTG

>spacer4

TGCCCGTTCTGCCTCTTCGCACTCTCGATCAA

>spacer5

TGCGTAATGGGCTACCTGAACTTCACATATCC

>spacer6

CGTATTCGTCACACAGCCCCGTCCAGAAATGA

>spacer7

TAACGAACTGAATAAAATGTCAGAAAGTGACG

>spacer8

GAAACGTAAACAGGGTAAGATACAACTCTGCA

>spacer9

TGTAAAGGGTGGTCTGGAAGGGGATCGGCAAA

**H133060377**

>spacer1

TTTTCAGCCCTTGTCGACTGCGGAACGCCCCT

>spacer2

GCGAAATAGTGGGGAAAAACCCCTGGTTAACC

>spacer3

TAGGCCTTGATACCATCGCTCGCACCTCGTCA

>spacer4

GTTTATTACTGCTTAGTTAATTAATGGGTTGC

>spacer5

AGGCGAATAATCTCTAATAGTCTCAATTCGTT

>spacer6

TAAATCTGGCGTCGAGACATTCGAAATAGTGC

>spacer7

TCTTTTGATTTTGCTGCGATGTTATAACCAGA

>spacer8

TATCCACATATACCCGCAATCATATTCAAGAA

>spacer9

AATCACTGCGGGGGTATTTAGCGGAAACGGCT

>spacer11

GCACCTCGAAACGGTTTTAAAACACTACCGTTT

>spacer12

TGGACCGATGGGGCCAACATCGCCGAACGTGG

>spacer13

GTTACGTTCGGTAAATGGAAAGCGGCGAATAT

>spacer14

CCAGAAAGTGCCGGTAGTGCCTGATGAACGAC

>spacer15

CGCGCCCACTTCCGTAAGATACAGATAATCCA

>spacer16

GGCAGCGGGCGAGGCAAACACATTCGGGGCGT

>spacer17

GGTAATTTCTCATCTAACAGCCTGTACGCCTC

>spacer18

GAATCTAATGCAACAGATGAATAAACACGTAA

>spacer19

TCTTTATCGTCAATGCGAAATTTTCCGCGACG

>spacer20

TCCCATTCACCAACAACAATATCGCCCTGCAA

>spacer21

ACTTCCTTCAGTCTTAACGATAATCCGCAACG

>spacer22

GCAAAATAGCGATGAGCTGGCTACGCCCACTGG

>spacer23

CAGCACGAAAAATTATTTACTGTCGTTGCTCA

>spacer1

ATCTTCATATTGCGTGACGCTGCCGATGAACG

>spacer2

TCTTTATCAGCTAACCATTTCCAGAACTCGTC

>spacer3

TATAATATGAATTAATTTTTGCGCATAACCTG

>spacer4

TGCCCGTTCTGCCTCTTCGCACTCTCGATCAA

>spacer5

TGCGTAATGGGCTACCTGAACTTCACATATCC

>spacer6

CGTATTCGTCACACAGCCCCGTCCAGAAATGA

>spacer7

TAACGAACTGAATAAAATGTCAGAAAGTGACG

>spacer8

GAAACGTAAACAGGGTAAGATACAACTCTGCA

>spacer9

TGTAAAGGGTGGTCTGGAAGGGGATCGGCAAA

**H133060378**

>spacer1

TTTGCCGATCCCCTTCCAGACCACCCTTTACA

>spacer2

TGCAGAGTTGTATCTTACCCTGTTTACGTTTC

>spacer3

CGTCACTTTCTGACATTTTATTCAGTTCGTTA

>spacer4

TCATTTCTGGACGGGGCTGTGTGACGAATACG

>spacer5

GGATATGTGAAGTTCAGGTAGCCCATTACGCA

>spacer6

TTGATCGAGAGTGCGAAGAGGCAGAACGGGCA

>spacer7

CAGGTTATGCGCAAAAATTAATTCATATTATA

>spacer8

GACGAGTTCTGGAAATGGTTAGCTGATAAAGA

>spacer9

CGTTCATCGGCAGCGTCACGCAATATGAAGAT

>spacer1

TGAGCAACGACAGTAAATAATTTTTCGTGCTG

>spacer2

CCAGTGGGCGTAGCCAGCTCATCGCTATTTTGC

>spacer3

CGTTGCGGATTATCGTTAAGACTGAAGGAAGT

>spacer4

TTGCAGGGCGATATTGTTGTTGGTGAATGGGA

>spacer5

CGTCGCGGAAAATTTCGCATTGACGATAAAGA

>spacer6

TTACGTGTTTATTCATCTGTTGCATTAGATTC

>spacer7

GAGGCGTACAGGCTGTTAGATGAGAAATTACC

>spacer8

ACGCCCCGAATGTGTTTGCCTCGCCCGCTGCC

>spacer9

TGGATTATCTGTATCTTACGGAAGTGGGCGCG

>spacer10

GTCGTTCATCAGGCACTACCGGCACTTTCTGG

>spacer11

ATATTCGCCGCTTTCCATTTACCGAACGTAAC

>spacer12

CCACGTTCGGCGATGTTGGCCCCATCGGTCCA

>spacer13

AAACGGTAGTGTTTTAAAACCGTTTCGAGGTGC

>spacer15

AGCCGTTTCCGCTAAATACCCCCGCAGTGATT

>spacer16

TTCTTGAATATGATTGCGGGTATATGTGGATA

>spacer17

TCTGGTTATAACATCGCAGCAAAATCAAAAGA

>spacer18

GCACTATTTCGAATGTCTCGACGCCAGATTTA

>spacer19

AACGAATTGAGACTATTAGAGATTATTCGCCT

>spacer20

GCAACCCATTAATTAACTAAGCAGTAATAAAC

>spacer21

TGACGAGGTGCGAGCGATGGTATCAAGGCCTA

>spacer22

GGTTAACCAGGGGTTTTTCCCCACTATTTCGC

>spacer23

AGGGGCGTTCCGCAGTCGACAAGGGCTGAAAA

**H133260293**

>spacer1

TTTGCCGATCCCCTTCCAGACCACCCTTTACA

>spacer2

TGCAGAGTTGTATCTTACCCTGTTTACGTTTC

>spacer3

CGTCACTTTCTGACATTTTATTCAGTTCGTTA

>spacer4

TCATTTCTGGACGGGGCTGTGTGACGAATACG

>spacer5

GGATATGTGAAGTTCAGGTAGCCCATTACGCA

>spacer6

TTGATCGAGAGTGCGAAGAGGCAGAACGGGCA

>spacer7

CAGGTTATGCGCAAAAATTAATTCATATTATA

>spacer8

GACGAGTTCTGGAAATGGTTAGCTGATAAAGA

>spacer9

CGTTCATCGGCAGCGTCACGCAATATGAAGAT

>spacer1

TGAGCAACGACAGTAAATAATTTTTCGTGCTG

>spacer2

CCAGTGGGCGTAGCCAGCTCATCGCTATTTTGC

>spacer3

CGTTGCGGATTATCGTTAAGACTGAAGGAAGT

>spacer4

TTGCAGGGCGATATTGTTGTTGGTGAATGGGA

>spacer5

CGTCGCGGAAAATTTCGCATTGACGATAAAGA

>spacer6

TTACGTGTTTATTCATCTGTTGCATTAGATTC

>spacer7

GAGGCGTACAGGCTGTTAGATGAGAAATTACC

>spacer8

ACGCCCCGAATGTGTTTGCCTCGCCCGCTGCC

>spacer9

TGGATTATCTGTATCTTACGGAAGTGGGCGCG

>spacer10

GTCGTTCATCAGGCACTACCGGCACTTTCTGG

>spacer11

ATATTCGCCGCTTTCCATTTACCGAACGTAAC

>spacer12

CCACGTTCGGCGATGTTGGCCCCATCGGTCCA

>spacer13

AAACGGTAGTGTTTTAAAACCGTTTCGAGGTGC

>spacer15

AGCCGTTTCCGCTAAATACCCCCGCAGTGATT

>spacer16

TTCTTGAATATGATTGCGGGTATATGTGGATA

>spacer17

TCTGGTTATAACATCGCAGCAAAATCAAAAGA

>spacer18

GCACTATTTCGAATGTCTCGACGCCAGATTTA

>spacer19

AACGAATTGAGACTATTAGAGATTATTCGCCT

>spacer20

GCAACCCATTAATTAACTAAGCAGTAATAAAC

>spacer21

TGACGAGGTGCGAGCGATGGTATCAAGGCCTA

>spacer22

GGTTAACCAGGGGTTTTTCCCCACTATTTCGC

>spacer23

AGGGGCGTTCCGCAGTCGACAAGGGCTGAAAA

**H133300609**

>spacer1

TTTGCCGATCCCCTTCCAGACCACCCTTTACA

>spacer2

TGCAGAGTTGTATCTTACCCTGTTTACGTTTC

>spacer3

CGTCACTTTCTGACATTTTATTCAGTTCGTTA

>spacer4

TCATTTCTGGACGGGGCTGTGTGACGAATACG

>spacer5

GGATATGTGAAGTTCAGGTAGCCCATTACGCA

>spacer6

TTGATCGAGAGTGCGAAGAGGCAGAACGGGCA

>spacer7

CAGGTTATGCGCAAAAATTAATTCATATTATA

>spacer8

GACGAGTTCTGGAAATGGTTAGCTGATAAAGA

>spacer9

CGTTCATCGGCAGCGTCACGCAATATGAAGAT

>spacer1

TGAGCAACGACAGTAAATAATTTTTCGTGCTG

>spacer2

CCAGTGGGCGTAGCCAGCTCATCGCTATTTTGC

>spacer3

CGTTGCGGATTATCGTTAAGACTGAAGGAAGT

>spacer4

TTGCAGGGCGATATTGTTGTTGGTGAATGGGA

>spacer5

CGTCGCGGAAAATTTCGCATTGACGATAAAGA

>spacer6

TTACGTGTTTATTCATCTGTTGCATTAGATTC

>spacer7

GAGGCGTACAGGCTGTTAGATGAGAAATTACC

>spacer8

ACGCCCCGAATGTGTTTGCCTCGCCCGCTGCC

>spacer9

TGGATTATCTGTATCTTACGGAAGTGGGCGCG

>spacer10

GTCGTTCATCAGGCACTACCGGCACTTTCTGG

>spacer11

ATATTCGCCGCTTTCCATTTACCGAACGTAAC

>spacer12

CCACGTTCGGCGATGTTGGCCCCATCGGTCCA

>spacer13

AAACGGTAGTGTTTTAAAACCGTTTCGAGGTGC

>spacer15

AGCCGTTTCCGCTAAATACCCCCGCAGTGATT

>spacer16

TTCTTGAATATGATTGCGGGTATATGTGGATA

>spacer17

TCTGGTTATAACATCGCAGCAAAATCAAAAGA

>spacer18

GCACTATTTCGAATGTCTCGACGCCAGATTTA

>spacer19

AACGAATTGAGACTATTAGAGATTATTCGCCT

>spacer20

GCAACCCATTAATTAACTAAGCAGTAATAAAC

>spacer21

TGACGAGGTGCGAGCGATGGTATCAAGGCCTA

>spacer22

GGTTAACCAGGGGTTTTTCCCCACTATTTCGC

>spacer23

AGGGGCGTTCCGCAGTCGACAAGGGCTGAAAA

**H133400611**

>spacer1

TTTGCCGATCCCCTTCCAGACCACCCTTTACA

>spacer2

TGCAGAGTTGTATCTTACCCTGTTTACGTTTC

>spacer3

CGTCACTTTCTGACATTTTATTCAGTTCGTTA

>spacer4

TCATTTCTGGACGGGGCTGTGTGACGAATACG

>spacer5

GGATATGTGAAGTTCAGGTAGCCCATTACGCA

>spacer6

TTGATCGAGAGTGCGAAGAGGCAGAACGGGCA

>spacer7

CAGGTTATGCGCAAAAATTAATTCATATTATA

>spacer8

GACGAGTTCTGGAAATGGTTAGCTGATAAAGA

>spacer9

CGTTCATCGGCAGCGTCACGCAATATGAAGAT

>spacer1

TGAGCAACGACAGTAAATAATTTTTCGTGCTG

>spacer2

CCAGTGGGCGTAGCCAGCTCATCGCTATTTTGC

>spacer3

CGTTGCGGATTATCGTTAAGACTGAAGGAAGT

>spacer4

TTGCAGGGCGATATTGTTGTTGGTGAATGGGA

>spacer5

CGTCGCGGAAAATTTCGCATTGACGATAAAGA

>spacer6

TTACGTGTTTATTCATCTGTTGCATTAGATTC

>spacer7

GAGGCGTACAGGCTGTTAGATGAGAAATTACC

>spacer8

ACGCCCCGAATGTGTTTGCCTCGCCCGCTGCC

>spacer9

TGGATTATCTGTATCTTACGGAAGTGGGCGCG

>spacer10

GTCGTTCATCAGGCACTACCGGCACTTTCTGG

>spacer11

ATATTCGCCGCTTTCCATTTACCGAACGTAAC

>spacer12

CCACGTTCGGCGATGTTGGCCCCATCGGTCCA

>spacer13

AAACGGTAGTGTTTTAAAACCGTTTCGAGGTGC

>spacer15

AGCCGTTTCCGCTAAATACCCCCGCAGTGATT

>spacer16

TTCTTGAATATGATTGCGGGTATATGTGGATA

>spacer17

TCTGGTTATAACATCGCAGCAAAATCAAAAGA

>spacer18

GCACTATTTCGAATGTCTCGACGCCAGATTTA

>spacer19

AACGAATTGAGACTATTAGAGATTATTCGCCT

>spacer20

GCAACCCATTAATTAACTAAGCAGTAATAAAC

>spacer21

TGACGAGGTGCGAGCGATGGTATCAAGGCCTA

>spacer22

GGTTAACCAGGGGTTTTTCCCCACTATTTCGC

>spacer23

AGGGGCGTTCCGCAGTCGACAAGGGCTGAAAA
